# Identification of Fibroblast Growth Factor Receptor 3 (FGFR3) as a Protein Receptor for Botulinum Neurotoxin Serotype A (BoNT/A)

**DOI:** 10.1371/journal.ppat.1003369

**Published:** 2013-05-16

**Authors:** Birgitte P. S. Jacky, Patton E. Garay, Jérôme Dupuy, Jeremy B. Nelson, Brian Cai, Yanira Molina, Joanne Wang, Lance E. Steward, Ron S. Broide, Joseph Francis, K. Roger Aoki, Raymond C. Stevens, Ester Fernández-Salas

**Affiliations:** 1 Allergan, Department of Biological Sciences, Irvine, California, United States of America; 2 The Scripps Research Institute, Department of Molecular Biology, La Jolla, California, United States of America; University of Illinois, United States of America

## Abstract

Botulinum neurotoxin serotype A (BoNT/A) causes transient muscle paralysis by entering motor nerve terminals (MNTs) where it cleaves the SNARE protein Synaptosomal-associated protein 25 (SNAP25_206_) to yield SNAP25_197_. Cleavage of SNAP25 results in blockage of synaptic vesicle fusion and inhibition of the release of acetylcholine. The specific uptake of BoNT/A into pre-synaptic nerve terminals is a tightly controlled multistep process, involving a combination of high and low affinity receptors. Interestingly, the C-terminal binding domain region of BoNT/A, H_C_/A, is homologous to fibroblast growth factors (FGFs), making it a possible ligand for Fibroblast Growth Factor Receptors (FGFRs). Here we present data supporting the identification of Fibroblast Growth Factor Receptor 3 (FGFR3) as a high affinity receptor for BoNT/A in neuronal cells. H_C_/A binds with high affinity to the two extra-cellular loops of FGFR3 and acts similar to an agonist ligand for FGFR3, resulting in phosphorylation of the receptor. Native ligands for FGFR3; FGF1, FGF2, and FGF9 compete for binding to FGFR3 and block BoNT/A cellular uptake. These findings show that FGFR3 plays a pivotal role in the specific uptake of BoNT/A across the cell membrane being part of a larger receptor complex involving ganglioside- and protein-protein interactions.

## Author Summary

Botulinum neurotoxin serotype A (BoNT/A) is one of seven neurotoxins (BoNT/A-G), produced by the bacteria Clostridium botulinum that are both poisons and versatile therapeutics. These toxins enter motor neurons where they prevent the release of acetylcholine at the neuromuscular junction. The specific uptake of BoNT/A across the neuronal cell membrane is dependent on specific receptor interactions. Binding to high density ganglioside GT1b mediates the initial binding step and via a low affinity interaction concentrates BoNT/A on the cell surface. Once anchored in the membrane, lateral movements within the plasma membrane facilitate intermolecular interactions of BoNT/A with additional lower density but higher affinity protein receptors. Here we present data supporting the identification of Fibroblast Growth Factor Receptor 3 (FGFR3) as a high affinity receptor for BoNT/A. We show that BoNT/A binds to FGFR3 with high affinity and functions as an agonist ligand for FGFR3. The identification of this novel receptor for BoNT/A represents an important advance in the understanding of the mechanism of action of BoNT/A, especially on the initial steps of neuronal uptake, and can be the basis for the development of new specific countermeasures and new BoNT/A-based therapeutics.

**Highlights:**

▸ Recombinant H_C_/A binds to the two extra-cellular loops of FGFR3b with a K_D_∼15 nM ▸ Recombinant H_C_/A acts as an agonist ligand for FGFR3 ▸The level of BoNT/A uptake is dependent on FGFR3 expression ▸ FGFR3 is expressed in motor nerve terminals

## Introduction

Botulinum neurotoxin serotype A (BoNT/A) is produced by *Clostridium botulinum* and is a member of the Clostridial neurotoxin family that includes BoNT/A-G and Tetanus neurotoxin (TeNT). BoNT/A causes transient muscle paralysis by entering motor nerve terminals (MNTs) where it cleaves nine amino acids from the C-terminus of the soluble N-ethylmaleimide-sensitive factor attachment receptor (SNARE) protein SNAP25 (SNAP25_206_) to yield SNAP25_197_ [1]. Intact SNAP25 is required for neurotransmitter release and cleavage of SNAP25 disrupts exocytosis, which blocks neurotransmitter release [2]–[5]. BoNT/A has become a useful pharmacological and biological tool. Because of its high potency and specificity for pre-synaptic nerve terminals, BoNT/A at picomolar concentrations, is used to treat a wide range of neuromuscular disorders [6]–[8], pain disorders including migraine [9], and excessive sweating [10]. The key to the exceptional specificity of BoNT/A is believed to be the mechanism of uptake across the presynaptic membrane of neurons that involves a combination of low and high affinity interactions known as the double receptor model [11]–[13].

The low affinity receptor for BoNT/A is the ganglioside GT1b with a binding pocket within the C-terminal portion of the receptor binding domain [12], [14], [15]. According to the APR receptor model [13], an array of presynaptic receptors (APRs), clustered in microdomains at the presynaptic membrane, are responsible for specific uptake of neurotoxins, including BoNT/A. It is the binding to high density ganglioside GT1b that mediates the initial binding step and via a low affinity interaction concentrates BoNT/A on the cell surface. GT1b has been shown to bind BoNT/A with a K_D_∼200 nM in vitro [16]. Once anchored in the membrane, lateral movements within the plasma membrane facilitate intermolecular interaction of BoNT/A with additional lower density but higher affinity protein receptors, including the three isoforms of Synaptic Vesicle (SV) glycoprotein 2, SV2A (ENSG00000159164), B (ENSG00000185518) and C (ENSG00000122012) that are exposed on the outer plasma membrane after fusion of synaptic vesicles to the presynaptic membrane [17]–[22]. BoNT/A specifically recognizes the fourth luminal domain (LD4) of SV2 [17], [18]. The specific sequence in the BoNT/A binding domain that interacts with SV2 has not been identified [23]. Glycosylated SV2A, B, and C have also been identified as receptors for BoNT/F [22], [24] and glycosylated SV2A and B have been identified as receptors for BoNT/E [20]. BoNT/D was reported to enter neurons via two ganglioside binding sites, one site at a position previously identified in BoNT/A, B, E, F, and G, and the other site resembling the second ganglioside-binding pocket of TeNT [25]. Recently, BoNT/D has also been shown to use SV2 (all three isoforms) to enter hippocampal neurons, but BoNT/D bound SV2 via a mechanism distinct from BoNT/A and E [26]. Surprisingly, SV2A and SV2B have also been reported to mediate binding and entry of TeNT into central neurons [27].

**Figure 1:**
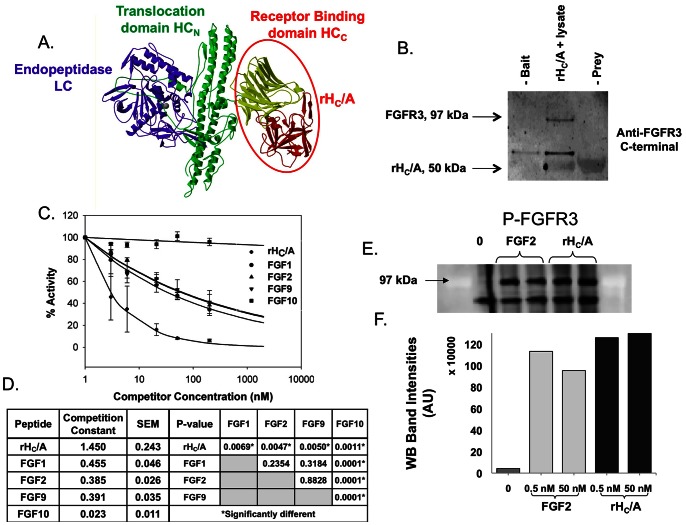
BoNT/A binds to FGFR3 and acts as an agonist ligand for FGFR3. (A) Crystal structure of Botulinum Neurotoxin Serotype A [28]. BoNT/A is a 150 kDa di-chain molecule with an approximately 50 kDa light chain (LC) and an approximately 100 kDa heavy chain (HC) held together by a single disulfide bond and non-covalent interactions. rH_C_/A is a 50 kDa recombinant protein consisting of only the HC_C_/A binding domain of BoNT/A (circle). (B) His-tag pull-down using His-rH_C_/A as “Bait” and differentiated PC-12 cell lysates as “Prey”. Samples were analyzed by Western blot, using antibody against FGFR3. A 97 kDa FGFR3 band was specifically pulled down by His-rH_C_/A. Excess amounts of His-rH_C_/A (150 µg) were used as “Bait” to capture FGFR3 from the cell lysate. The bound protein was released from the beads by addition of 2× SDS-PAGE loading buffer. The final sample therefore contains large amounts of His-rH_C_/A to FGFR3 and the His-rH_C_/A is seen as a 50 kDa unspecific “ghost” band on the final Western blot. Negative controls samples without either “Bait” or “Prey” were run in parallel. Results from an additional pull-down experiment using biotin transfer to pull down the receptor from intact Neuro-2a cells are shown in Figure S1C, D, and E. (C) BoNT/A cell-based competition assay demonstrating that native ligands for FGFR3; FGF1, FGF2, and FGF9 were able to compete with BoNT/A for binding to the receptor. Neuro-2a cells were pre-treated for 30 min with increasing concentrations of FGF1, FGF2, FGF9 (native ligands for FGFR3) or FGF10 (not a ligand for FGFR3, negative control) before treatment with 1 nM BoNT/A. In parallel, cells were incubated with rH_C_/A (positive control). SNAP25 cleavage, as a measure for BoNT/A uptake and activity, was decreased when cells were pre-incubated with rH_C_/A, FGF1, FGF2, or FGF9. No decrease was observed after pre-incubation with FGF10. Data from a minimum of 4 experiments are included. (D) The data was fitted to a non-linear exponential decay model; Y = 100*e- CC*log(concentration). As a measure for competition, the Competition Constant (CC) and standard error (SE) for each competitor are shown. The three native ligands for FGFR3: FGF1, FGF2, and FGF9 competed equally well. There was no significant difference between the CC values of FGF1, FGF2, and FGF9, P-value≥0.2354. The CC value for FGF10 was significant lower than the other CC values, P-value≤0.0011, and the CC value for rH_C_/A was higher than the other CC values, P-value≤0.0069. (E–F) Immunoprecipitation (IP) and Western Blot assay, using anti-phosphotyrosine conjugated beads to capture phosphorylated proteins and Anti-FGFR3 to identify and visualize FGFR3 among these captured phosphorylated proteins. The IP was performed with cells that were either kept untreated (negative control) (E:Lane 1), or treated, for 10 minutes, with either 0.5 (E:Lane 2) or 50 nM (E:Lane 3) FGF2 (positive control), or 0.5 (E:Lane 4) or 50 nM (E:Lane 5) rH_C_/A. (F) Graphic presentation of the calculated Western blot band intensities. An increase in phosphorylated FGFR3, ∼30-fold, was observed after treatment with either FGF2 (Light grey) or rH_C_/A (Black), compared to the untreated control (Gray). There was no difference in phosphorylated FGFR3 between the 0.5 and 50 nM concentration of either FGF2 or rH_C_/A.

Analysis of the first crystallographic structure of BoNT/A revealed a ganglioside binding site with structural homology to that within TeNT [28], [29](Figure 1A). A potential protein binding site was also identified with structural homology to basic fibroblast growth factor (FGFb or FGF2; ENSG00000138685), agglutinin, and the toxin abrin. The report of a potential FGF2 protein binding site within BoNT/A served as the basis for the identification of FGFR3 (Fibroblast Growth Factor Receptor 3, ENSG00000068078) as a receptor for BoNT/A. FGFR3 [30], [31] is one of four receptor-tyrosine kinases (FGFR1–4) that act as receptors for FGFs. FGFRs are composed of an extra-cellular ligand-binding domain consisting of three immunoglobulin-like loops (L1–L3), a transmembrane domain, and a split cytoplasmic tyrosine kinase domain. FGFRs are activated by dimerization induced by ligand binding that enables the cytoplasmic kinase domains to transphosphorylate one another at specific tyrosine residues [32]–[35]. FGFR1–3, but not FGFR4, exist in three different splice variants that differ in the C-terminal half of L3 and determine their individual ligand affinity and specificity. The splice variants are referred to as “a”, “b”, and “c” [36]–[39]. The “b” and “c” variants are expressed on the cell surface, while the “a” splice variant, which lacks the transmembrane domain [40], becomes a secreted extra-cellular FGF-binding protein [41]. Among the 22 known FGFs, FGF1 (ENSG00000113578), members of the subfamilies FGF8 (FGF8, 17, 18) and FGF9 (FGF9; ENSG00000102678, 16, 20) have been shown to function as ligands for both FGFR3b and c but with different levels of affinity. FGF2, and the subfamilies FGF4 (FGF4, 5, 6) and FGF19 (FGF19, 21, 23) have been shown to function as ligands for FGFR3c [39], [42]. Moreover, FGFs bind their receptors in the presence of one or more low affinity co-factors including heparin sulfate (HS), gangliosides, neuropilin-1, Klotho, and anosmin that function to modulate receptor activity [43]–[48].

The studies presented in this manuscript identify FGFR3 expressed in motor neurons at MNTs as a functional protein receptor for BoNT/A. The C-terminal binding domain of BoNT/A, H_C_/A, binds to the second and third extra-cellular ligand binding domain of FGFR3 and results in the phosphorylation of FGFR3. It is demonstrated that cellular uptake of BoNT/A is dependent on the level of FGFR3 expression. Native ligands for FGFR3; FGF1, FGF2, and FGF9 compete for binding to FGFR3 and block BoNT/A uptake in a cell-based assay. Moreover, peptides derived from the FGFR3 subtype b and c extra-cellular domain block BoNT/A uptake in neuronal cells. Both FGFR3 subtype b and c bind to rH_C_/A, but FGFR3b has the highest affinity with a K_D_∼15 nM in vitro. These data suggest that FGFR3 is a potential high affinity component of a receptor complex for BoNT/A on the presynaptic membrane.

## Results

### Identification of FGFR3 as a functional receptor for BoNT/A

Analysis of the BoNT/A crystal structure (Figure 1A) revealed that the H_C_/A subdomain has structural homology to basic fibroblast growth factor (FGF) [28]. To investigate the interaction of BoNT/A and FGFR, pull-down assays were performed with Neuro-2a and PC-12 cell lines that have been shown to take up BoNT/A with high efficacy after differentiation by serum starvation and trophic factors, and Nerve Growth Factor (NGF) for PC-12 cells [49], [50]. Figure S1A shows a representative experiment of how differentiation increases BoNT/A uptake in PC-12 cells. BoNT/A uptake was determined by treatment of cells with BoNT/A, followed by incubation and Western blot analysis of the SNAP25_197_ cleavage product. After optimization of differentiation and treatment conditions, differentiated Neuro-2a and PC-12 cells were treated with BoNT/A and EC_50_ values of 60±5 pM and 47.1±13 pM, respectively were determined (Figure S1B).

A complex containing BoNT/A and its receptor was isolated using three alternative pull-down methods, the results from two of these methods are shown here. First, Sulfo-SBED BoNT/A was used in a biotin transfer experiment to pull down the receptor from intact Neuro-2a cells. Antibodies against FGFR3 detected a band of the correct molecular weight for FGFR3 as part of a 250 kDa complex with BoNT/A. Figure S1C, D, and E demonstrate that a 250 kDa protein complex was isolated and that specific bands for BoNT/A and FGFR3 within this complex could be detected using antibodies specific to BoNT/A or FGFR3. Second, a complex containing both BoNT/A and FGFR3 was isolated from Neuro-2a cells treated with biotin labeled BoNT/A and the cross linking reagent Bis(Sulfosuccinimidyl) suberate (data not shown). Finally, the recombinant binding domain of BoNT/A, His-rH_C_/A, was used to pull down FGFR3 from differentiated PC-12 cell lysates without the use of cross-linking reagents, demonstrating a strong interaction (Figure 1B).

Having identified FGFR3 as a binding partner for BoNT/A, we investigated the role of FGFR3 as a functional receptor for BoNT/A. Competition experiments utilizing native ligands for FGFR3; FGF1, FGF2, and FGF9 [39], [42], demonstrated that these ligands competed for binding to FGFR3 and blocked BoNT/A uptake in a cell-based assay with differentiated Neuro-2a cells (Figure 1C–D). rH_C_/A, was used as a positive control and produced a strong blockade of BoNT/A uptake. As a negative control, FGF10 (ENSG00000070193), which is not a ligand for FGFR3, but closely related to the other FGF ligands tested, was used. Pre-incubation with FGF10 did not affect BoNT/A uptake. The experimental data from at least four independent experiments were compiled and fitted to a non-linear exponential decay model; Y = 100*e- CC*log(concentration). The Competition Constant (CC) for each fitted curve was calculated and demonstrated similar competition of the three FGF ligands. The data strongly suggest that BoNT/A utilizes FGFR3 to gain entry into neuronal cells since native FGFR3 ligands blocked its uptake. The hypothesis that BoNT/A acts as an agonist for FGFR3 was further supported by demonstrating that treatment with rH_C_/A resulted in phosphorylation of FGFR3, achieving similar levels of activation as cells treated with identical concentrations of FGF2 (Figure 1E–F).

### FGFR3b Loop 2,3 binds to rH_C_/A with high affinity

**Figure 2:**
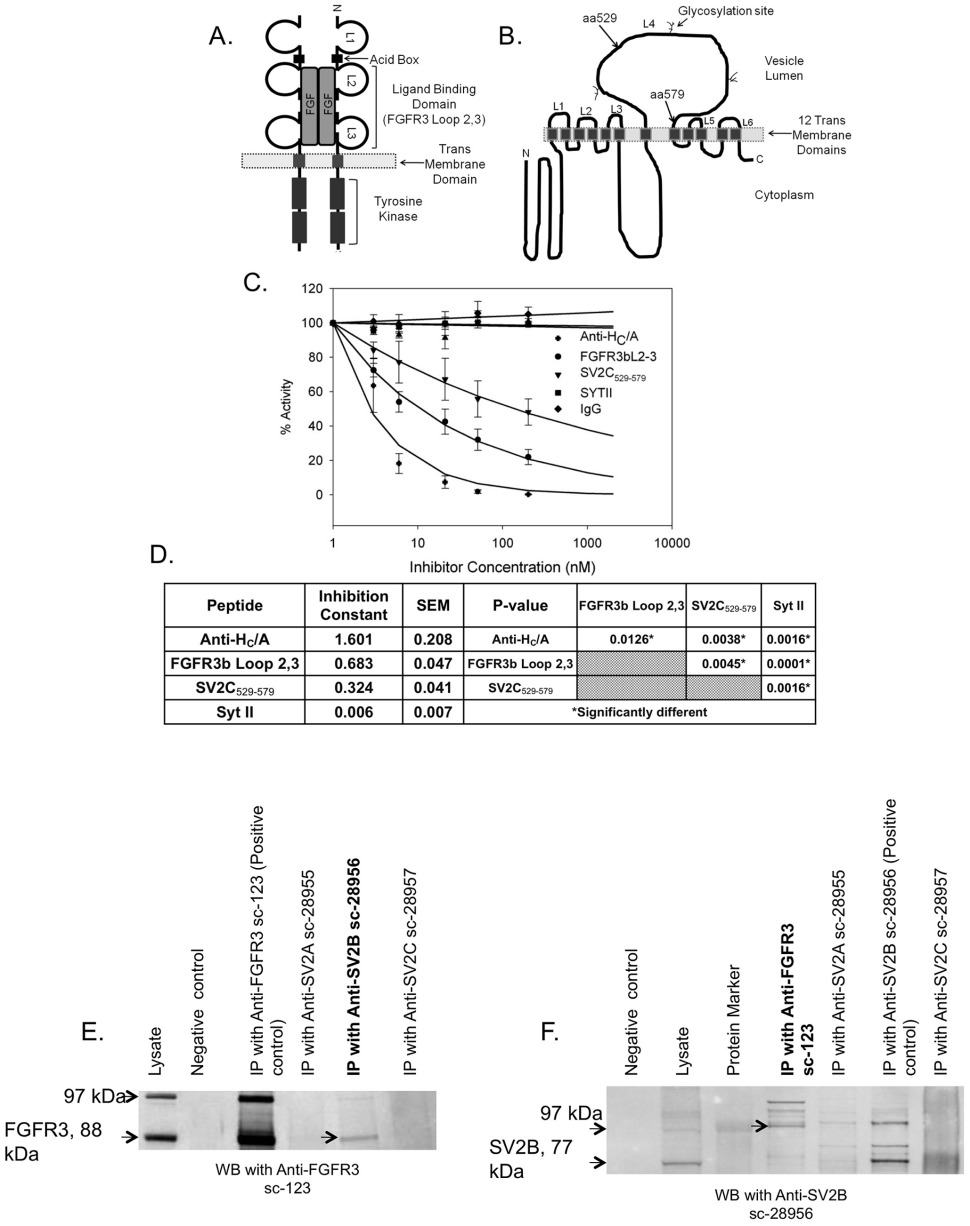
FGFR3b Loop 2,3 and SV2C_529–579_ block BoNT/A uptake in a cell-based assay. (A) Schematic presentation of FGFR3 and the FGFR3b Loop 2,3 peptide consisting of the second and third loops of FGFR3b that have previously been identified as the ligand binding domain [39], [52]. (B) Schematic presentation of SV2C and the peptide SV2C_529–579_. SV2C is a 12 transmembrane vesicular protein that contains three N-glycosylation sites within the LD4 luminal domain and it is highly glycosylated in vivo [20], [71], [72]. The red arrows mark the 6 kDa peptide, SV2C_529–579_, which spans part of the LD4 luminal domain of SV2C. (C) Pre-incubation of BoNT/A with FGFR3b Loop 2,3 or SV2C_529–579_ inhibit BoNT/A uptake in Neuro-2a cells. BoNT/A, at 1 nM concentration, was incubated for 20 min with increasing concentrations of FGFR3b Loop 2,3 or SV2C_529–579_ before treatment of cells. In parallel, BoNT/A was incubated with neutralizing antibodies to H_C_/A (positive control), or with Syt II_1–20_ (negative control). Data are shown as the percentage inhibition compared to inhibition after addition of Anti-H_C_/A. SNAP25 cleavage, as a measure of BoNT/A uptake, decreased when BoNT/A was pre-incubated with Anti- H_C_/A, FGFR3b Loop 2,3, or SV2C_529–579_. No inhibition was observed after addition of Syt II_1–20_. The averages of three or more experiments are included. (D) Data was fitted to a non-linear exponential decay model; Y = 100*e- IC*log(concentration). As a measure for inhibition, the Inhibition Constant (IC) and standard error (SE) for each inhibitor are shown. The IC values are all significantly different from each other, P-value≤0.0045. (E–F) Protein Complex Immunoprecipitation (Co-IP) and Western blot (WB). IP's were performed with antibody to FGFR3 (sc-123), SV2A (sc- 28955), SV2B (sc-28956), and SV2C (sc-28957). WB's were performed with antibody to FGFR3 (sc-123) (E) and SV2B (sc-28956) (F). FGFR3 and SV2 are glycosylated in vivo and multiple bands are detected on WB. The predicted MW for non-glycosylated protein is 88 kDa for FGFR3 and 77 kDa for SV2B. The positive control, IP and WB with the same antibody, showed clear bands on both blots and the negative control (antibody only, no lysate) showed no bands. An interaction between FGFR3 and SV2 was detected, in both directions, using the Anti-SV2B (sc-28956) antibody that recognizes SV2B and, to a lesser extent, SV2A and SV2C.

The ligand binding site for FGFR3 has been identified as the second and third extra-cellular loops of FGFR3 (Figure 2A) [39], [51], [52]. To further verify the functional role of FGFR3 as a receptor for BoNT/A, we demonstrated that pre-incubation of BoNT/A with a peptide spanning the second and third extra-cellular loops of FGFR3b (FGFR3b Loop 2,3) inhibited BoNT/A uptake presumably via binding to the receptor binding domain of BoNT/A (Figure 2C). Inhibition, although to a lesser extent, was also observed using the peptide spanning the luminal domain (LD4) of SV2C, SV2C_529–579_ (Figure 2C). SV2C_529–579_ (Figure 2B) has previously been reported as the minimal peptide region for binding to BoNT/A [17]. As a positive control for inhibition, BoNT/A was pre-incubated with a neutralizing monoclonal antibody directed to the binding domain of BoNT/A, Anti-H_C_/A. As a negative control, Synaptotagmin II (aa1–20, Syt II_1–20_), the receptor for BoNT/B [53]–[56] was used. The experimental data from at least three independent experiments were compiled and fitted to a non-linear exponential decay model; Y = 100*e- IC*log(concentration). The Inhibition Constant (IC) for each fitted curve was calculated and demonstrated that FGFR3b Loop 2,3 inhibited BoNT/A uptake and was a more effective uptake inhibitor than SV2C_529–579_ (Figure 2D).

Initial experiments designed to explore the combined effect of FGFR3b Loop 2,3 and SV2C_529–579_ showed that the peptides bound with good affinity in vitro (data not shown). To address the question as to whether FGFR3 and SV2 interact in neurons, we performed a series of Co-IPs experiments. We tested if an antibody to FGFR3 could pull-down SV2 isoforms from a differentiated Neuro-2a cell lysate, and vice versa, antibodies to SV2 isoforms could pull-down FGFR3. An interaction between FGFR3 and SV2 was detected using the Anti-SV2B (sc-28956) antibody, which recognizes SV2B and, to a lesser extent, SV2A and SV2C. No bands were detected when using antibodies for SV2A or SV2C (data not shown). The result suggests that FGFR3 and SV2B interact in differentiated Neuro-2a cells (Figure 2E–F).

**Figure 3:**
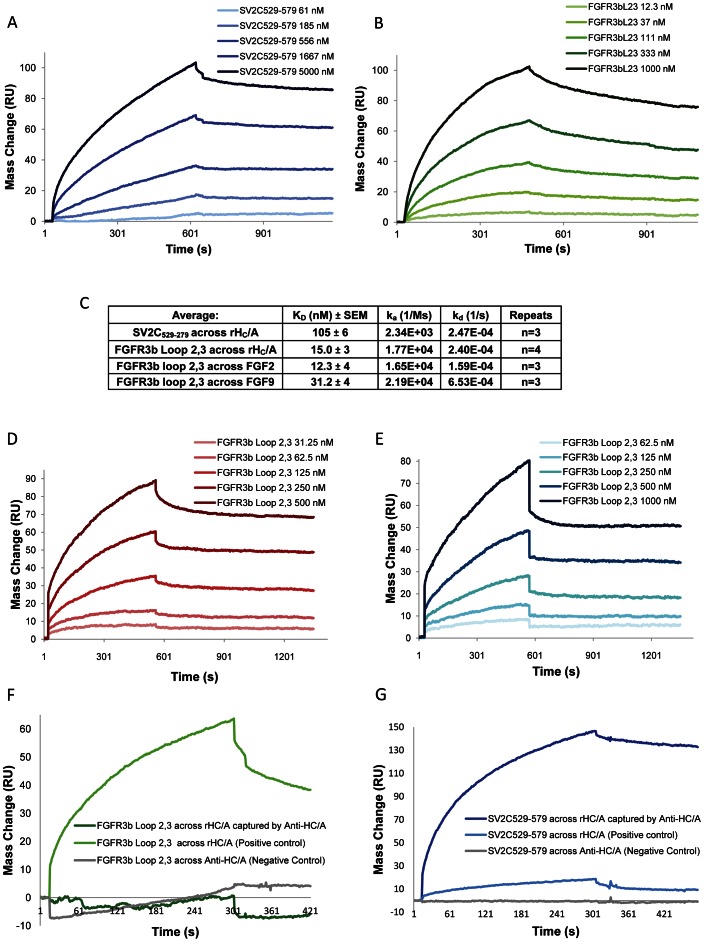
FGFR3b Loop 2,3 binds to rH_C_/A in vitro with high affinity compared to SV2C_529–579_ and the affinity is similar to the binding affinity of the native ligands FGF2 and FGF9. (A) BIAcore SPR Binding Affinity measurements of SV2C_529–579_ binding to rH_C_/A. Shown here are sensorgrams from a single experiment, including the binding of increasing concentrations of SV2C_529–579_: 21, 61, 185, 556, 1667, and 5000 nM respectively to a CM5 sensor chip covered with rH_C_/A (∼3000 RUs). The curves were fitted to a 1∶1 kinetic-binding model (A + B ↔ AB) with the BIAevaluation 3.0 software and the binding constants; the association constant (k_a_), dissociation constant (k_d_), and the binding affinity at equilibrium (K_D,_ = K_d_/K_a_ (at equilibrium)) were estimated. The binding constants for the experiment shown were estimated to; K_D_ = 96.2 nM, k_a_ = 1.29E+03 1/Ms, k_d_ = 1.24E-04 1/s. (B) BIAcore SPR Binding Affinity measurements of FGFR3b Loop 2,3 binding to rH_C_/A. Shown here are sensorgrams from a single experiment, including the binding of increasing concentrations of FGFR3b Loop 2,3: 12, 37, 111, 333, and 1000 nM respectively to a CM5 sensor chip covered with rH_C_/A (∼3000 RUs). The curves were fitted to a 1∶1 kinetic-binding model (A + B ↔ AB) with the BIAevaluation 3.0 software and the binding constants; k_a_, k_d_, and K_D,_ = K_d_/K_a_ (at equilibrium) were estimated. The binding constants for the experiment shown were estimated to; K_D_ = 15.1 nM, k_a_ = 1.73E+04 1/Ms, k_d_ = 2.62E-04 1/s. (C) Table showing the binding constant averages for SV2C_529–579_ upon binding to rH_C_/A (n = 3) and for FGFR3b Loop 2,3 upon binding to rH_C_/A (n = 4), FGF2 (n = 3), or FGF9 (n = 3). (D) BIAcore SPR Binding Affinity measurements of FGFR3b Loop 2,3 binding to FGF2. Shown here are sensorgrams from a single experiment, including the binding of increasing concentrations of FGFR3b Loop 2,3: 31.25, 62.5, 125, 250, and 500 nM respectively to a CM5 sensor chip covered with FGF2. The curves were fitted to a 1∶1 kinetic-binding model (A + B ↔ AB) with the BIAevaluation 3.0 software and the binding constants; k_a_, k_d_, and K_D,_ = K_d_/K_a_ (at equilibrium) were estimated. The binding constants for this experiment were estimated to; K_D_ = 13.5 nM, k_a_ = 4.98E+03 1/Ms, k_d_ = 6.69E-05 1/s. (E) BIAcore SPR Binding Affinity measurements of FGFR3b Loop 2,3 binding to FGF9. Shown here are sensorgrams from a single experiment, including the binding of increasing concentrations of FGFR3b Loop 2,3: 31.25, 62.5, 125, 250, 500, and 1000 nM respectively to a CM5 sensor chip covered with FGF9. The curves were fitted to a 1∶1 kinetic-binding model (A + B ↔ AB) with the BIAevaluation 3.0 software and the binding constants; k_a_, k_d_, and K_D,_ = K_d_/K_a_ (at equilibrium) were estimated. The binding constants for this experiment were estimated to; K_D_ = 29.9 nM, k_a_ = 2.19E+03 1/Ms, k_d_ = 6.53E-05 1/s. (F–G) Dual binding experiment: 1000 nM FGFR3b Loop 2,3 (F) or 3000 nM SV2C_529–579_ (G) were flowed across rH_C_/A (∼1000 RU) captured by anti-H_C_/A 6B1 monoclonal antibody (3000 RU). The result show that SV2C_529–579_ and anti-H_C_/A antibody can bind simultaneously to rH_C_/A, while FGFR3b Loop 2,3 binding is abolished. Background drift from dissociating rH_C_/A was subtracted. As a negative control, the peptides were flowed across anti-H_C_/A antibody before capture of rH_C_/A. As a positive control, the peptides were flowed across a surface with ∼1000 RU immobilized rH_C_/A in parallel. Upon immobilization by amine coupling the protein is randomly oriented, in contrast to what occurs when the protein is captured by antibody, where the protein is uniformly oriented. This means that the number of free binding sites differs despite equal amounts of ligand protein used in the experiment. This can be seen as a larger binding response for SV2C_529–579_ flowed across captured rH_C_/A compared to amine-immobilized rH_C_/A. These data suggest that the anti-H_C_/A monoclonal antibody binds to an epitope close to or at the same site as FGFR3b Loop2,3 and that FGFR3b Loop 2,3 and SV2C_529–579_ recognize the binding domain of BoNT/A via two distinct binding sites.

To characterize the binding of FGFR3 and SV2C to rH_C_/A, the binding affinity of the two receptor surrogate peptides, FGFR3b Loop 2,3 and SV2C_529–579_ to rH_C_/A was tested in a Surface Plasmon Resonance (SPR) binding assay. FGFR3b Loop 2,3 bound to rH_C_/A with an average K_D_ = 15.0±3 nM, n = 4, k_a_ = 1.77E+04 1/Ms, k_d_ = 2.40E-04 1/s (Figure 3B–C). This is similar to what has been reported earlier upon binding of FGFR2b Loop 2,3 to FGF2, K_D_ = 12.8±0.3 nM [57]. SV2C_529–579_ bound to rH_C_/A with an average K_D_ = 105±6 nM, n = 3, k_a_ = 2.34E+03 1/Ms, k_d_ = 2.47E-04 1/s (Figure 3A,C). The difference in affinity between FGFR3b Loop 2,3 and SV2C_529–579_ is due to a 10 times faster association, k_a_ is estimated to be 10 times higher for FGFR3b Loop 2,3 versus SV2C_529–579_. It can be seen on the curves as a more shallow slope and longer time to equilibrium for SV2C versus FGFR3b Loop 2,3 (Figure 3A versus 3B).

In order to compare the binding affinity of FGFR3b Loop 2,3 to rH_C_/A to the binding affinity of native ligands for FGFR3, the binding affinity of FGFR3b Loop 2,3 to FGF2 and FGF9 was also measured in the SPR binding assay. FGFR3b Loop 2,3 bound to FGF2 with an average K_D_ = 12.3±4 nM, n = 3, k_a_ = 1.65E+04 1/Ms, K_d_ = 1.59E-04 1/s. FGFR3b Loop 2,3 bound to FGF9 with an average K_D_ = 31.2±1 nM, n = 3, k_a_ = 2.92E+03 1/Ms, k_d_ = 9.25E-05 1/s (Figure 3C–E).

Having identified rH_C_/A as an agonist ligand for FGFR3 (Figure 1E–F) and shown that rH_C_/A binds to FGFR3b Loop 2,3 in vitro with similar affinity as native ligands for FGFR3, we evaluated if FGFR3 would facilitate uptake of rH_C_/A and native ligands in a similar fashion. We utilized HEK 293 cells as a model system, because they express FGFR3 but no measurable levels of any of the SV2 isoforms (Figure S1G). Consequently, uptake of rH_C_/A via SV2 should be absent in these cells. HEK 293 cells do not express SNAP25 and therefore SNAP25 cleavage could not be used as a measure for BoNT/A uptake. Instead, uptake was measured as an increase in intracellular fluorescence after addition of fluorescently labeled rH_C_/A or FGF2, a native ligand for FGFR3. The results showed slightly less uptake of rH_C_/A compared to FGF2, but similar kinetics (Figure S1H). The slightly higher uptake of FGF2 compared to rH_C_/A could be due to FGF2 having more receptor targets, since it is a general ligand for FGFRs. These data suggest that FGFR3 can mediate BoNT/A uptake independently of SV2.

To explore the binding sites of FGFR3b Loop 2,3 and SV2C_529–579_ on the binding domain of BoNT/A, we performed a series of dual binding experiments using the BIAcore. We tested if the peptides and anti-H_C_/A, the neutralizing monoclonal antibody previously used in the cell based inhibition assay (Figure 2C & D), could bind to rH_C_/A simultaneously. rH_C_/A was captured by anti-H_C_/A monoclonal and FGFR3b Loop 2,3 or SV2C_529–579_ were flowed across. The results show that binding of anti-H_C_/A monoclonal blocks binding of FGFR3b Loop 2,3, but not SV2C_529–579_ to rH_C_/A in vitro (Figure 3F–G), demonstrating that FGFR3 and SV2 bind to different sites on the BoNT/A binding domain. Interestingly, these data also suggest that inhibition of BoNT/A uptake by the neutralizing monoclonal anti-H_C_/A antibody in the cell based assay is due to blockage of FGFR3 binding.

These results demonstrate that FGFR3b Loop 2,3 and SV2C_529–579_ can both inhibit the activity of BoNT/A in a cell-based assay, but FGFR3b Loop 2,3 is a stronger inhibitor than SV2C_529–579_ . They show that in an *in vitro* binding assay, the binding affinity for FGFR3b Loop 2,3 upon binding to rH_C_/A is higher, due to an estimated 10 times faster association, than the binding affinity for SV2C_529–579_ upon binding to rH_C_/A. The binding affinity for FGFR3b Loop 2,3 to rH_C_/A, is similar or identical, to the binding affinity for FGFR3b Loop 2,3 upon binding to FGF2 and FGF9, two native ligands for FGFR3. Also, uptake of rH_C_/A in HEK 293 cells, that express FGFR3, but not SV2, is comparable to uptake of FGF2, supporting a case for uptake of BoNT/A via FGFR3 independent of the presence of SV2. Finally, dual in vitro binding studies using a neutralizing antibody to H_C_/A, show that the FGFR3 and SV2C peptides bind to rH_C_/A at different sites, FGFR3 at a site close to or overlapping the binding site for the anti- H_C_/A, and SV2C in a site distal from the anti- H_C_/A binding site. Different binding sites for FGFR3 and SV2 would allow a multi-receptor complex to form.

### FGFR3 expression levels affect the sensitivity of cells to BoNT/A

Differentiation of neuronal cells increases BoNT/A uptake (Figure S1A and B). It has been suggested that the increased sensitivity in differentiated PC-12 cells is due to increased expression of the SNAP25b subtype that is most sensitive to BoNT/A [50]. Since the increased sensitivity could also be a result of increased expression of a receptor for BoNT/A, we studied the expression of FGFR3 as well as SV2A, B, and C before and after differentiation in both Neuro-2a and PC-12 cells. FGFR3 expression levels were similar in both cell lines and the amount of FGFR3 was unchanged after differentiation. Neuro-2a cells expressed mostly SV2C, while PC-12 cells expressed all three SV2 isoforms. Surprisingly, differentiation resulted in decreased expression of SV2 isoforms in both cell lines (Figure S1F).

**Figure 4:**
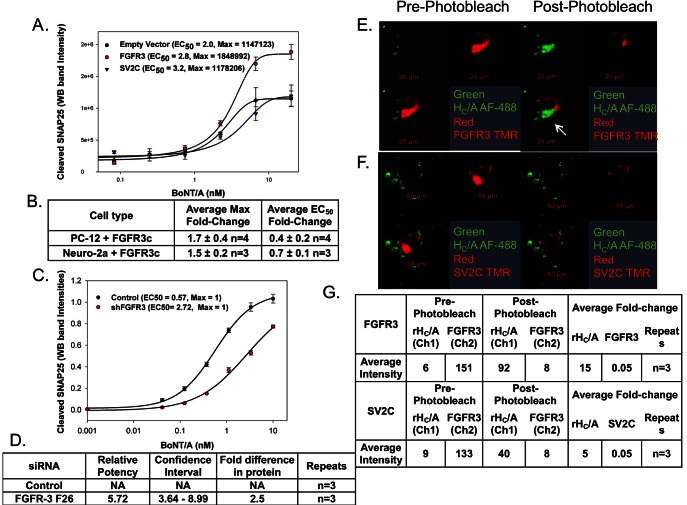
Binding to FGFR3 is a rate-liming step for BoNT/A uptake in neuronal cells under resting conditions. (A) PC-12 cells transfected with FGFR3c, SV2C, or empty vector were treated with 0–30 nM BoNT/A for 6 hours, and the amount of cleaved SNAP25 was detected by Western blot. (A) The dose response curve for one representative experiment is shown. Cells transfected with FGFR3c had a higher maximum signal (higher efficacy) than the cells transfected with SV2C or empty vector (pcDNA3.1). (B) Table showing the average fold increase in maximal signal response and the average fold decrease in EC_50_ after transfection of PC-12 or Neuro-2a cells with FGFR3c, compared to cells transfected with empty vector (See also Figure S2) . (C) shRNA knockdown of FGFR3. Dose response curves of PC-12 cells stably transfected with Scrambled (negative control) or FGFR3 shRNAs and then treated with 0–30 nM BoNT/A for 6 hours. The curves were plotted as cleaved SNAP25 (SNAP25_197_) vs. BoNT/A concentration. Cells transfected with shFGFR3 were less sensitive to BoNT/A, the EC_50_ was ∼5-fold higher, compared to cells transfected with random control shRNA. (D) Table showing the average fold difference in relative potency. The relative potency is calculated by dividing the potency of a test sample with the potency of a reference sample. A relative potency of 1 means that both samples are identical. If the test sample is more potent than the reference the relative potency is smaller than 1. If the sample is less potent than the reference, then the relative potency is higher than one. In this case, cells transfected with FGFR3 shRNA are 5.7-fold less sensitive than cells transfected with scrambled shRNA (See also Figure S2). (E–F) Photobleaching experiment where the recovery of AF-488 labeled rH_C_/A (donor) fluorescence was measured after photobleaching of either TMR labeled FGFR3 (E) or TMR labeled SV2C (acceptor). (G) Table showing the fluorescence intensity values before and after photobleaching for three separate experiments.

Assuming FGFR3 is a functional receptor for BoNT/A, one would expect overexpression of FGFR3 to result in increased binding of BoNT/A on the cell membrane. If receptor binding is a rate-limiting step, this should also result in increased sensitivity to BoNT/A. Experiments to test the sensitivity to BoNT/A were performed under non-depolarizing conditions, where the exposure of SV2 on the cell surface is presumed to be limited. Overexpression of FGFR3 in PC-12 and Neuro-2a cells increased the sensitivity to BoNT/A and produced higher efficacy (increased maximal signal), in a Western blot SNAP25_197_ cell-based assay, while overexpression of SV2C did not (Figure 4A–B).

FGFR3 overexpression also increased binding of transfected cell membranes to rH_C_/A in a SPR binding assay, while overexpression of SV2C did not (Figure S2A and C), suggesting that if there is more FGFR3 on the cell surface more BoNT/A will bind, while more SV2C does not increase BoNT/A binding. Human neuroblastoma SH-SY5Y cells were also evaluated in the SPR binding assay because they have low sensitivity to BoNT/A [58] and express very little endogenous SV2C. Even in this situation, there was no effect as a result of over expressing SV2C (Figure S2B).

We also demonstrated, utilizing shRNA, that reduced expression of FGFR3 resulted in reduced sensitivity to BoNT/A. A 65% reduction of FGFR3 protein expression resulted in a 5.7-fold decrease in potency and a ∼5-fold increase in EC_50_ when compared to control cells (Figure 2C). No change in the protein expression levels of either SV2A, B, or C was detected in those samples. A separate experiment with siRNAs for FGFR3 and SV2C demonstrated that a 4.2-fold reduction in SV2C mRNA resulting in a 2-fold reduction in protein levels did not cause a reduction in BoNT/A uptake (Figure S2E–F). While a 3-fold reduction of FGFR3 mRNA resulting in a 2-fold reduction in protein levels reduced sensitivity to BoNT/A causing a 3-fold shift in relative potency when compared to control cells (Figure S2D and F), confirming that, under non-depolarizing conditions, binding to FGFR3 is a rate-limiting step in BoNT/A uptake.

The interaction between FGFR3 and rH_C_/A was also observed in a photobleaching experiment using the FRET partners AF-488 and TMR. We detected an increase in the fluorescent signal from the AF-488 labeled rH_C_/A (donor) after photobleaching the TMR labeled FGFR3 (acceptor). The data shows that FGFR3 and rH_C_/A are proximal enough within PC-12 cells to FRET, suggesting that FGFR3 not only binds BoNT/A on the cell surface, but it is also trafficking with BoNT/A within the cells. There was little change in the fluorescence observed when the experiment was performed with TMR labeled SV2C as the acceptor (Figure 4E–G).

### FGFR3 is expressed at rat Motor Nerve Terminals

**Figure 5:**
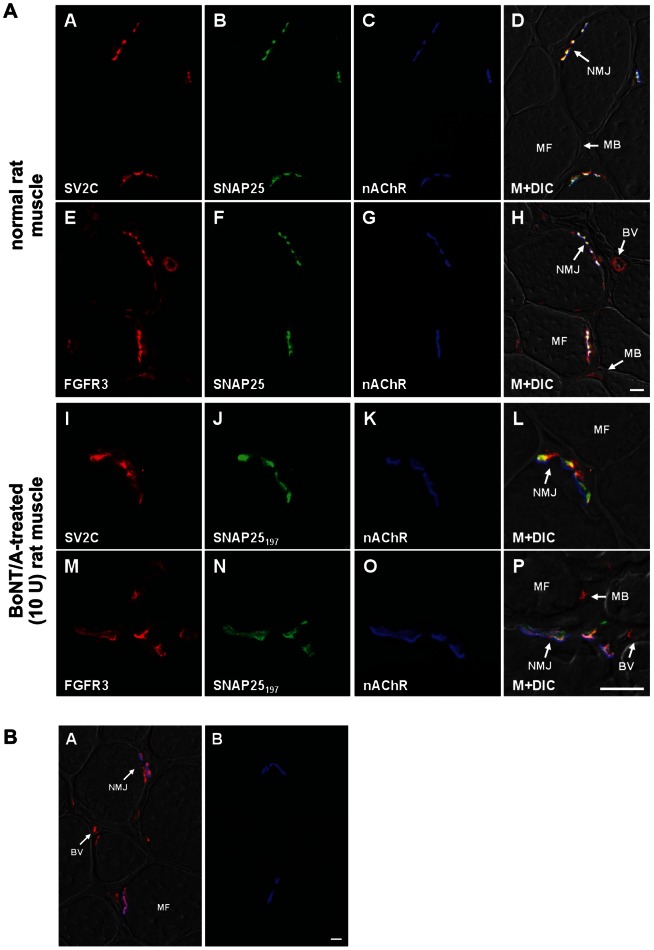
FGFR3 and SV2C are both expressed on motor nerve terminals in rat skeletal muscle. (A) Photomicrographs from normal rat TA muscle cross-sections (A–H) and from muscles pre-treated with 10 U of BOTOX (I–P). Sections show immunostaining for SV2C (red, A, I), FGFR3 (red, E, M), SNAP25 (green, B, F) and SNAP25_197_ (green, J, N). Nicotinic acetylcholine receptors (nAChR) are labeled by α-bungarotoxin Alexa-Fluor 647 (blue, C, G, K, O). The last column shows a merge of the staining patterns within that row on a muscle fiber background imaged by DIC optics (M+DIC). BV, Blood Vessel; MB, Myoblast; MF, myofiber; NMJ, neuromuscular junction. Note the overlap in IR signal for FGFR3 and SV2C with SNAP25_197_ and directly adjacent to the pattern of post-synaptic nAChR expression. Scale bar = 10 µm in H (A–H) and P (I–P). (B) A. Photomicrograph from a saline-treated rat TA muscle cross-section showing immune-staining for FGFR3 (red) co-localized with α-bungarotoxin-labeled nAChRs (blue). No staining was observed for SNAP25_197_ (green) in these sections. B. A normal rat muscle cross-section immune-stained with peptide-quenched FGFR3 antibodies and lacking the SNAP25 primary antibody shows only α-Bgt-labeled nAChRs. Scale bar = 10 µm. BV, Blood Vessel; MF, myofiber; NMJ, neuromuscular junction.

BoNT/A causes transient muscle paralysis through presynaptic blockade of acetylcholine release at the neuromuscular junction. If FGFR3 functions as a receptor for BoNT/A *in vivo*, then it would be reasonable to presume that FGFR3 should be expressed at the MNTs. The expression pattern of the FGFR3 receptor was examined on cross-sections of rat skeletal muscle to look for potential co-expression with SV2C, SNAP25, and nicotinic acetylcholine receptors (nAChRs). Overall, immuno-reactive (IR) staining for SV2C and SNAP25 were co-expressed exclusively at neuromuscular junctions (NMJs) throughout the muscle (Figure 5A, A–D). These NMJs were specifically defined by using fluorescently labeled α-bungarotoxin (α-Bgt) nAChRs. In contrast, FGFR3-IR was not only detected at NMJs, but also in extra-synaptic structures, such as myoblasts and blood vessels (Figure 5A, E). At the NMJs however, the FGFR3 staining pattern corresponded to that of SNAP25 and nAChRs (Figure 5A, E–H). To verify expression of SV2C and FGFR3 within BoNT/A sensitive NMJs, we treated rat Tibialis Anterior (TA) muscles with BoNT/A and analyzed the staining patterns for SV2C and FGFR3 together with IR-staining for cleaved SNAP25 (SNAP25_197_). Focusing on individual synapses, we observed overlapping patterns for SV2C-IR and SNAP25_197_-IR that were adjacent to the pattern of post-synaptic nAChR expression (Figure 5A, I-L). Similarly, the patterns for FGFR3-IR and SNAP25_197_-IR at the NMJ were overlapping and appeared adjacent to the pattern of nAChR expression (Figure 5A, M–P). Saline-treated rat muscles showed no immuno-staining for SNAP25_197_ (Figure 5B). These qualitative results demonstrate that FGFR3 receptors are present on MNTs and are co-expressed with SV2C and SNAP25.

### The second and third extra-cellular loop of FGFR3 is the minimal optimal binding site for H_C_/A

**Figure 6:**
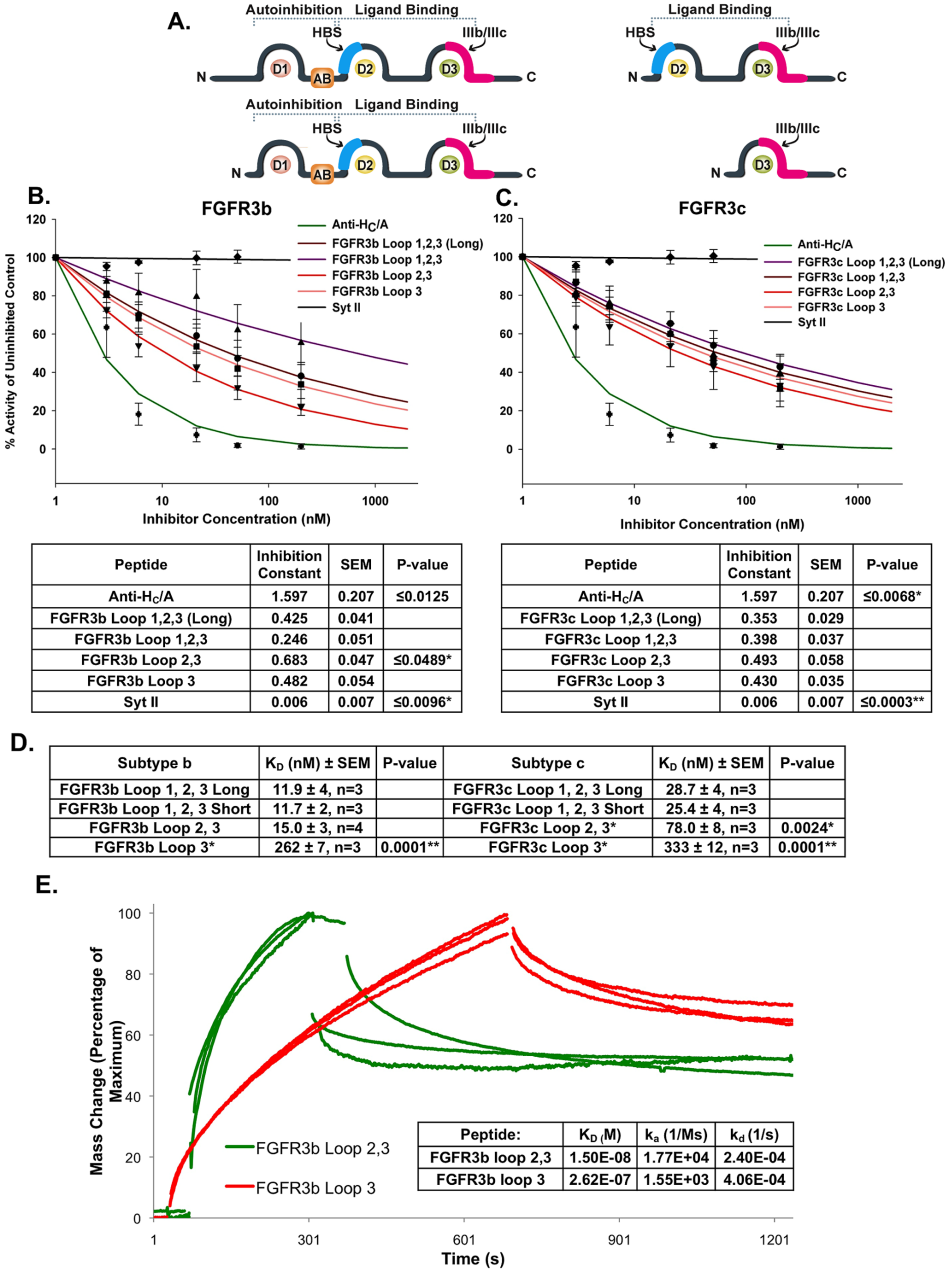
BoNT/A binds to FGFR3 Loop 2,3 with the highest affinity *in vitro*. (A) Schematic presentation of the eight deletion mutant FGFR3 peptides. FGFR3b Loop 1,2,3 and FGFR3c Loop 1,2,3 (long and short version); FGFR3b Loop 2,3 and FGFR3c Loop 2,3; and FGFR3b Loop 3 and FGFR3c Loop 3. AB: Acid Box; HBS: Heparin Binding Site. The purple area highlights the region where subtype b and c differ in sequence (Figure modified from [73] (See also Figure S3)). (B–C) Pre-incubation of BoNT/A with eight deletion mutant FGFR3 peptides, corresponding to the extra-cellular loops of FGFR3b/c (A) inhibit BoNT/A uptake in Neuro-2a cells. BoNT/A at 1 nM was incubated for 20 min with increasing concentrations of individual FGFR3 peptides before treatment of cells. In parallel, BoNT/A was pre-incubated with antibodies to H_C_/A (positive control), or Syt II_1–20_ (negative control). Data are shown as percentage BoNT/A uptake relative to the uptake after pre-incubation with the negative control Syt II_1–20_. SNAP25 cleavage, as a measure of BoNT/A uptake, was decreased when BoNT/A was pre-incubated with Anti-H_C_/A or with any of the eight deletion mutant FGFR3 peptides. The averages of three or more experiments were included. The data was fitted to a non-linear exponential decay model; Y = 100*e- IC*log(concentration). As a measure for inhibition, the Inhibition Constant (IC) and standard error (SE) for each inhibitor are shown. The IC value for Syt II_1–20_ was significantly lower than all the other IC values, p≤0.0096 and p≤0.0003 respectively. The IC value for Anti-H_C_/A was significantly higher than all the other IC values, p≤0.0125 and p≤0.0068 respectively. The IC value for FGFR3b Loop 2,3 was significantly higher than the IC values for the other FGFR3b peptides, p-value≤0.0489. There was no significant difference in IC value among the FGFR3c peptides. (D) Results from testing the eight FGFR3 deletion mutant peptides in a BIAcore SPR binding assay. Increasing concentrations, 0–4000 nM of each of the deletion mutant FGFR3 peptides were flowed across a CM5 sensor chip covered with rH_C_/A. The curves were fitted to a 1∶1 kinetic-binding model (A + B ↔ AB) with the BIAevaluation 3.0 software. The table shows the calculated K_D_'s ± SEM of all eight FGFR3 deletion mutant peptides upon binding to rH_C_/A. The average k_a_'s (association constants (1/Ms)) and k_d_'s (dissociation constants (1/s)) are shown in figure S3B. The K_D_ values for the FGFR3b Loop 1,2,3 peptides are significantly lower than the K_D_ values for the FGFR3c Loop 1,2,3 peptides, P-value≤0.0005 and the K_D_ values for FGFR3b/c Loop 3 and FGFR3c Loop 2,3 are significantly higher than the K_D_ values for the longer FGFR3b/c peptides, P-value≤0.0024. Loop 2,3 of FGFR3 can therefore be identified as the minimal optimal binding region of FGFR3 (See also Figure S3). (E) Comparison of FGFR3b loop 2,3 and FGFR3b Loop 3 association (or on-rate) after normalization. The 1000 nM curves of three different runs were normalized using BIAcore evaluation software version 4.1. The on-rate for FGFR3b loop 2,3 was faster than the on-rate for FGFR3b Loop 3. The average k_a_ (1/Ms), k_d_ (1/s), and K_D_ (K_D_ = k_d_/k_a_ (M)) are shown. k_a_ for FGFR3b Loop 2,3 is ∼10-fold higher than k_a_ for FGFR3b Loop 3 (See also Figure S3).

We have shown that FGFR3b Loop 2,3 binds to BoNT/A with low nanomolar affinity. To further identify the binding site for BoNT/A and to test whether the two subtypes of FGFR3, subtype b and c, bound with similar affinities, we constructed eight deletion mutants of FGFR3, containing either FGFR3 Loop 1,2,3 (long or short version), Loop 2,3, or Loop 3 of both subtypes (Figure 6A). The difference between FGFR3b and FGFR3c lies in the most C-terminal part of Loop 3 (Figure S3A). All the deletion mutant FGFR3 peptides were able to inhibit BoNT/A uptake in a cell-based inhibition assay, presumably via binding to the receptor binding domain of BoNT/A and preventing binding to cells (Figure 6B–C). However, in a SPR binding assay a significantly lower affinity was observed for the peptides spanning only Loop 3 compared to the peptides spanning Loop 2,3 or Loop 1,2,3. The association (on-rate) of the peptides spanning only Loop 3 was ∼10 times lower than the on-rate of the longer peptides, while the dissociation (off-rate) was similar (Figure 6E and S3B–C). The similar off-rate can explain why the peptides are able to inhibit equally well BoNT/A binding to the receptor in the cell-based inhibition assay. In the cell-based assay sufficient time (20 min) is available for even slow associating peptides to bind and the ability to inhibit relies more on a slow dissociation. In the SPR binding assay, on the other hand, binding is observed in real time (5–10 min). Based on the lower on-rate of the Loop 3 peptides observed in the SPR binding assay, Loop 2,3, which is the binding region for native FGF ligands, was identified as the minimal optimal binding region for BoNT/A. FGFR3b bound with slightly higher affinity than FGFR3c (Figure 6D).

## Discussion

In this study we identified FGFR3 as a high affinity protein receptor for BoNT/A. Pull-down experiments with neuronal cells resulted in the identification of a protein complex containing BoNT/A and FGFR3. Native ligands for FGFR3; FGF1, FGF2, and FGF9 compete with rH_C_/A for binding to the receptor and binding of rH_C_/A results in phosphorylation of FGFR3, demonstrating that BoNT/A acts as an agonist ligand for FGFR3. Since ligand binding and activation of FGFRs are known to result in receptor-mediated endocytosis of both receptor and ligand [59], we propose that binding of BoNT/A to FGFR3 also results in endocytosis and that FGFR3 may mediate BoNT/A uptake in both stimulation independent and stimulation dependent manners. This hypothesis is supported by the fact that depolarization of nerve cells increases uptake (stimulation dependent), while at the same time BoNT/A uptake can take place in resting neurons (stimulation independent) [3], [4], [60]–[63]. This is also supported by the observation that the uptake, but not the initial binding step, is altered by nerve stimulation [64].

Motor neurons at MNTs take up BoNT/A with high affinity, resulting in inhibition of exocytosis and muscle paralysis. Thus, MNTs should presumably express a BoNT/A receptor(s), and our results clearly demonstrated that both FGFR3 and SV2C are present at MNTs. These data support the hypothesis that FGFR3 functions as a high affinity receptor for BoNT/A uptake, and that most likely, SV2 is only available as a receptor for BoNT/A after depolarization and vesicular exocytosis.

Using a SPR binding assay, we demonstrated that a peptide spanning the second and third extra-cellular loops of FGFR3, FGFR3b Loop 2,3, binds to rH_C_/A with a K_D_∼15 nM and that a peptide spanning the luminal domain of SV2C, SV2C_529–579_, binds to rH_C_/A with a K_D_∼100 nM *in vitro*. The observed ∼15 nM affinity for binding of rH_C_/A to FGFR3b Loop 2,3 was similar or identical to the affinity for binding of two native ligands for FGFR3, FGF2 and FGF9 to FGFR3b Loop 2,3 in the same assay. Also, comparable uptake of rH_C_/A and FGF2 was observed in HEK 293 cells, a cell line that express FGFR3 and not any of the SV2 isoforms, suggesting that FGFR3 can mediate uptake of BoNT/A independently of SV2.

A recent publication [63] clearly supports our findings. The authors observed limited co-localization of SV2C and H_C_/A or BoNT/A after treatment of spinal cord motor neurons under resting conditions and this co-localization did not significantly increase under depolarizing conditions. Moreover, inhibition of exocytosis by pre-treatment with BoNT/D did not prevent the internalization of H_C_/A. The authors concluded that BoNT/A may exploit an alternative pathway(s), largely independent of stimulated synaptic endo-exocytosis, to enter neuronal cells in both resting and depolarizing conditions.

Pre-incubation of BoNT/A with the FGFR3 and SV2C peptides before treatment of cells, blocked uptake in neuronal cells, presumably by interacting with the binding domain of BoNT/A and preventing binding to the receptor on cells. In accordance with the observed lower affinity for SV2C_529–579_ compared to Loop 2,3 of FGFR3, the FGFR3 peptide produced a stronger blockade than the SV2C peptide. These data suggest that FGFR3 may function as a high affinity receptor for BoNT/A and that SV2C may function as a medium affinity receptor.

SPR experiments demonstrated that the FGFR3b and SV2C peptides bind to different sites on H_C_/A, FGFR3 in a site overlapping the epitope of a neutralizing monoclonal antibody to H_C_/A (6B1, provided by Dr. L. Smith, USAMRIID) and SV2C in a site distal from both. So far the binding site for SV2 has not been identified, but it has been suggested that SV2 binds to the C-terminal half of the binding domain, H_CC_, similar to how Synaptotagmin II binds to BoNT/B [23]. Different binding sites for FGFR3 and SV2 on the binding domain of BoNT/A would allow formation of a multi-receptor complex. Interestingly, Co-IP experiments show that FGFR3 and SV2 can interact in live Neuro-2a cells, suggesting a step-wise binding and/or formation of a multi-receptor BoNT/A complex.

We concur with others in the field that the specificity of BoNT/A for neuronal cells and specially motor neurons, which is higher than binding to a single receptor can explain, is due to the fact that uptake of BoNT/A is a multi-step process involving at least two crucial steps [13], [23]. The first crucial step is binding to gangliosides like GT1b (KD∼200 nM) that are abundantly present in the outer leaflet of the plasma membrane of neuronal cells. This initial step increases the local concentration of BoNT/A and allows it to diffuse in the plane of the membrane to bind its protein receptor(s) [15], [16], similar to what has been observed for heparin sulfate and FGF2 [65]. BoNT/A that is diffusing within microdomains of the plasma membrane will be presented to FGFR3 and/or SV2, bind to the receptor, and undergo endocytosis representing a second crucial step.

There are several lines of evidence suggesting that the initial binding to gangliosides is critical to specifically accumulate BoNT/A on the membrane of neuronal cells. For example, It has been shown [66] that, in the absence of GT1b, Neuro-2a cells are insensitive to BoNT/A and that knockout mice defective in the production of polysialogangliosides show reduced sensitivity to BoNT/A and BoNT/B [20], [26], [67]. Moreover, a mutant version of H_C_/A, W1266L & Y1267S that does not bind to GT1b, does not extend paralysis time caused by BoNT/A in murine phrenic nerve-hemidiaphragm preparations demonstrating an impaired ability to bind to neuronal cells [68]. Here we propose that only after BoNT/A is anchored at the neuronal membrane the second crucial step, binding to FGFR3 and/or SV2, can occur. This explains how BoNT/A can specifically enter motor neurons by recognizing FGFR3, a receptor also expressed by non-neuronal cells that lack gangliosides in their membranes. As evidence for a second crucial step, we demonstrate that, in Neuro-2a cells, if either FGFR3 or SV2C binding is blocked, BoNT/A uptake is impaired.

BoNT/A uptake is affected by the cellular levels of FGFR3 expression. We demonstrated that overexpression of FGFR3 increased binding of membrane extracts to rH_C_/A as well as BoNT/A uptake in three different neuronal cell lines, while down-regulation of FGFR3 reduced uptake of BoNT/A. In contrast, no changes in BoNT/A uptake were observed when increasing or decreasing the expression of SV2C, suggesting that FGFR3, but not SV2C represents a rate-limiting step in BoNT/A uptake under resting conditions. This is consistent with our finding that FGFR3, but not SV2C, co-localized with BoNT/A in un-stimulated PC-12 cells.

By testing eight deletion mutant peptides of the FGFR3b and c extra-cellular domain in the SPR binding assay, we identified the extra-cellular Loop 2,3 of FGFR3 as the minimal optimal binding site for rH_C_/A. Native ligands for FGFR3 also bind to Loop 2,3 [39], [51], [52] and these data demonstrate that the binding site for rH_C_/A overlaps the binding site for native ligands of FGFR3. The affinity measurements also demonstrated that FGFR3b bound with slightly higher affinity than FGFR3c, the K_D_ for the subtype b peptides was ∼15 nM, while the K_D_ for the subtype c peptides was ∼25 nM. The FGFR3c subtype is the subtype expressed in the nervous system, while expression of the FGFR3b subtype is restricted to epithelial structures [69], [70]. It is therefore more likely that BoNT/A utilizes the FGFR3c subtype *in vivo* to gain access into neuronal cells.

In conclusion, this paper presents evidence for FGFR3 as a high affinity receptor for BoNT/A, potentially being part of a larger receptor complex involving sugar- and protein-protein interactions. FGFR3 is present in the target motor neurons. Overexpression of FGFR3 in several neuronal cells increases efficacy and sensitivity to BoNT/A while decreased FGFR3 expression renders the cells less sensitive. BoNT/A binds to FGFR3 at the same extra-cellular region and with the same affinity as native ligands for FGFR3 and functions as an agonist ligand inducing FGFR3 phosphorylation. Moreover, BoNT/A uptake can be blocked by native FGFR3 ligands or by peptide fragments containing the extra-cellular region of FGFR3. Together, these results expand our knowledge of BoNT/A uptake in neuronal cells and present a potential new pathway mediating BoNT/A entry and trafficking into neurons under both resting and depolarizing conditions.

## Materials and Methods

### Cell lines and growth conditions

*Unless otherwise stated tissue culture reagents were from Invitrogen (Carlsbad, CA)*

***PC-12-*** Rat pheochromocytoma cell line (CRL-1721; ATCC) was cultured in collagen IV plates (354528; BD). Growth media: RPMI media with 2 mM GlutaMAX, 5% Fetal Bovine Serum (heat-inactivated), 10% Equine Serum, 10 mM HEPES, 1 mM Sodium Pyruvate, 100 U/ml Penicillin, and 100 µg/ml Streptomycin. Differentiation media: RPMI media with 2 mM GlutaMAX, 1× B27 supplement, 1× N2 supplement, 10 mM HEPES, 1 mM Sodium Pyruvate, 50 ng/ml NGF, 100 U/ml Penicillin, and 100 µg/ml Streptomycin. ***Neuro-2a-*** Murine neuroblastoma cell line (CCL-131; ATCC) was cultured in Costar Tissue Culture Flasks (CLS3150; Corning). Growth media: EMEM with 2 mM GlutaMAX, 0.1 mM Non-Essential Amino-Acids, 10 mM HEPES, 1 mM Sodium Pyruvate, 100 U/ml Penicillin, 100 µg/ml Streptomycin, and 10% Fetal Bovine Serum. Differentiation media: EMEM with 2 mM GlutaMAX, 0.1 mM Non-Essential Amino-Acids, 10 mM HEPES, 1× N2 supplement, and 1× B27 supplement. ***SH-SY5Y-*** Human neuroblastoma cell line (94030304; ECACC) was cultured in Costar Tissue Culture Flasks (CLS3150; Corning). Growth media: EMEM with 2 mM GlutaMAX/F12, 0.1 mM Non-Essential Amino-Acids, 10 mM HEPES, 1 mM Sodium Pyruvate, 100 U/ml Penicillin, 100 µg/ml Streptomycin, and 10% Fetal Bovine Serum. Differentiation media: EMEM with 2 mM GlutaMAX, 0.1 mM Non-Essential Amino-Acids, 10 mM HEPES, 1× N2 supplement, and 1× B27 supplement. ***HEK 293-*** Human Embryonic Kidney 293 cells (CRL-1573; ATCC) were cultured in Costar Tissue Culture Flasks (CLS3150; Corning). Growth media: EMEM with 2 mM GlutaMAX, 0.1 mM Non-Essential Amino-Acids, 10 mM HEPES, 1 mM Sodium Pyruvate, 100 U/ml Penicillin, 100 µg/ml Streptomycin, and 10% Fetal Bovine Serum.

For differentiation, PC-12, Neuro-2a, and SH-SY5Y cells were plated in 96-well plates at 5×10^4^ cells/well in 100 µl differentiation media for three days.

### Affinity purification of rH_C_/A and FGFR3b/c peptides

FGFR3b/c peptides and rH_C_/A were expressed from pET-29 b (+) in *E.Coli*, Acella Electrocompetent BL21(DE3) (42649; Edge Biosystems). Expression was induced by 1 mM IPTG (V3955; Promega) at either 37°C for 16 hours (FGFR3b/c peptides) or at 16°C for 16 hours (rH_C_/A). For purification of rH_C_/A, the supernatant was collected after centrifugation and the protein was purified using the MagneHis Protein Purification System (V8500; Promega). FGFR3b/c peptides were purified from inclusion bodies. After expression, cells were first lysed for 1 hour in five times the cell wet weight in lysis buffer containing 50 mM Tris-HCl pH 8.0, 10 mM EDTA, 100 mM NaCl, 10 mM DTT, 5% (v/v) glycerol, protease inhibitor (P1860; Sigma), 150 mU/ml rLysozyme (71110; EMD Chemicals), and 50 mU/ml benzonase nuclease (70746; EMD Chemicals) and then sonicated for 5 minutes. Pellets were collected by centrifugation and washed three times, first time with wash buffer (50 mM Tris-HCl pH 8.0, 100 mM NaCl, 10 mM DTT, 5% glycerol and 2% Triton X-100) plus 10 mM EDTA, second time with wash buffer only, and third time with wash buffer plus 2 M urea. The inclusion bodies were dissolved in 50 mM Tris-HCl pH 8.0, 500 mM NaCl, 10 mM DTT, 8 M urea, 10 mM imidazole and the peptides were isolated using the Magne-His Protein Purification Resin (V8560; Promega). Wash buffer: 50 mM Tris-HCl pH 8.0, 500 mM NaCl, 10 mM DTT, 8 M urea, and 20 mM imidazole. Elution buffer: 50 mM Tris-HCl pH 8.0, 500 mM NaCl, 10 mM DTT, 8 M urea, and 500 mM imidazole. After elution, the buffer was exchanged to 50 mM Tris-HCl pH 8.0, 1 mM EDTA, 3.8 mM GSH, 1.2 mM GSSH and 1 M arginine using a FastDialyzer fitted with 5 kDa MWCO cellulose acetate membranes (Harvard Apparatus).

### His tag pull-down assay

The assay was performed according to the protocol from Pierce (21277; Pierce). 150 µg of rH_C_/A (26 mg/ml stock conc.) was used as “Bait” protein and 500 µl of differentiated PC-12 cell lysate (from 1.5×10^6^ cells) was used as “Prey” protein. As negative controls, samples without either “Bait” or “Prey” protein were run in parallel. The eluted samples were analyzed by SDS-PAGE and Western blot analysis.

### Pull-down using Sulfo-SBED (Biotin transfer)

10 µg BoNT/A was reacted with 2 mM Sulfo-SBED (33073; Thermo Scientific) (solubilized at 125 mM in DMSO) in 0.1 ml PBS for 2 hours. The reaction was stopped by addition of 0.1 µl 0.4 M Tris. As a control 10 µg of BSA was also reacted with 2 mM Sulfo-SBED. Sulfo-SBED BoNT/A and BSA were added to 1×10^8^ Neuro-2a cells and mixed by rotisserie at 4°C for 4 hours. The reagent was photoactivated for 15 minutes with a UV light source. The cells were washed 4 times with cold TBS and then lysed by incubation for 2 hours in T-X-100 lysis buffer (50 mM Tris, 150 mM NaCl, 1% Triton X-100, 10 mM EDTA, pH 7.2). The biotinylated proteins were precipitated using Monomeric Avidin (Thermo Scientific), washed 4 times in the TX100 lysis buffer and then analyzed by SDS-PAGE and Western Blot Analysis.

### SDS-PAGE and Western blot analysis

Samples were dissolved in 2× SDS-PAGE loading buffer (LC2676; Invitrogen), heated to 95°C for 10 min, resolved in 12% 26-well Criterion gels (345-019; Bio-Rad) or 12% Bis-Tris Novex NuPage gels (NP0341BOX; Invitrogen), transferred to 0.45 µm nitrocellulose membranes (62-0233; Bio-Rad), blocked for 1 hour in TBS buffer (170-6435; BioRad) plus 0.1% Tween 20 (161-0781; BioRad) (TBS-T) and 2% blocking agent (RPN418V; GE Healthcare), and incubated overnight with primary antibody, either; anti-SNAP25_197_ (Allergan) rabbit polyclonal antibody diluted to 1 µg/ml, anti-H_C_/A (Allergan) rabbit polyclonal antibody diluted to 1 µg/ml, anti-FGFR3 (1∶500, sc-123; Santa Cruz Biotechnology), anti-FGFR3 (1∶1000, Ab133644; Abnova), anti-SV2A, (1∶200, sc-28955; Santa Cruz Biotechnology), anti-SV2B (1∶200, sc-28956; Santa Cruz Biotechnology), anti-SV2C (1∶500, sc-28957; Santa Cruz Biotechnology) or anti-Syntaxin (1∶200, sc-12736; Santa Cruz Biotechnology) in TBS-T plus 2% blocking agent. Secondary antibody was anti-rabbit IgG H+L HRP conjugate (81-6120; Invitrogen), anti-rabbit IgG veriBlot for IP secondary antibody (HRP) (ab131366, Abcam) (used for IP only), and anti-mouse IgG H+L HRP conjugate (62-6520; Invitrogen) diluted 1∶5000 in TBS-T plus 2% blocking agent. Membranes were developed using ECL Plus Western Blotting System Detection Reagents (RPN2132; GE Healthcare). The Chemiluminescence was captured using a Typhoon 9140 (GE Healthcare) set to the following parameters: 455 nm excitation laser and detector set to all wavelengths below 520 nm emissions. The intensity of the gel bands were calculated using Image Quant software TL V2005 (GE Healthcare). The data was analyzed using PLA and SigmaPlot v 10.0 (Systat Software Inc.). Intensity values were plotted against concentration of BoNT/A in log scale and fitted to a 4-parameter logistics function (Y = Y_0_+a/[1+(X/X_0_)^b^]) without constraints. Based on the fitted curves the EC_50_ values, corresponding to “X_0_”, were determined.

### Transfection of cells and membrane extraction

Cells were transfected with pcDNA3.1 (+) (V790-20; Invitrogen), FGFR3c (EX-Y0098-M50; Genecopoeia), SV2C (EX-S2660-M050; Genecopoeia), RNAi Hi GC (12935-400; Invitrogen), FGFR3 siRNA-88 (FGFR3RSS331488; Invitrogen), FGFR3 siRNA-89 (FGFR3RSS331489; Invitrogen), SV2C-1 siRNA (AM16708; Ambion). Membrane extractions were performed with a Native Membrane Protein Extraction kit (444810; Calbiochem). Total protein concentration was measured using Bradford Reagent (500-0205; Bio-Rad).

### Immunoprecipitation of phosphorylated membrane proteins

Differentiated Neuro-2a cells transfected with FGFR3 were treated with 0.5 nM or 50 nM FGF2 (233-FB; R&D Systems) or rH_C_/A (26 mg/ml stock concentration) for 10 minutes. Membrane extracts were prepared and 100 µg of total protein was incubated with 40 µl of a 50% slurry of anti-phosphotyrosine conjugated beads (16-101; Millipore) for 24 hours at 4°C. Samples were washed 4 times with MEB buffer (50 mM Tris pH 7.5, 150 mM NaCl) containing phosphatase inhibitor cocktail 1 and 2 (P2850 and P5726; Sigma) and complete protease inhibitor cocktail (11 873 580 001; Roche) and analyzed by SDS-PAGE and Western blot analysis, using antibody against FGFR3.

### Treatment with BoNT/A

Differentiated cells (see **Cell Lines and Growth Conditions**) were treated with 0–30 nM BoNT/A (0.41 mg/ml stock concentration) in differentiation media for 6 or 24 hours followed by overnight or two day incubation in toxin-free media. Cell lysates were analyzed by SDS-PAGE and Western blot analysis, using antibody against cleaved SNAP25, anti-SNAP25_197_ (Allergan).

### BoNT/A cell-based competition/inhibition assays

For competition, before treatment with 1 nM BoNT/A (150 kDa, Metabiologics), Neuro-2a cells were pre-treated for 30 min with increasing concentrations of FGF1, FGF2, FGF9, FGF10 (negative control) (132-FA; 233-FB; 273-F9, and 345-FG; R&D Systems), or rH_C_/A (Figure 1A, positive control). For inhibition, before treatment onto cells, 1 nM BoNT/A (150 kDa, Metabiologics) was incubated for 20 min with increasing concentrations of either; FGFR3b/c deletion mutant peptides (Figure 6A), SV2C_529–579_ (JPT Peptide Technologies; aa529–279, H-NTYFKNCTFIDTVFDNTDFEPYKFIDSEFKNCSFFHNKTGCQITFDDDYSA-NH2, Figure 2B), monoclonal anti-H_C_/A 6B1 (Provided by Dr. L. Smith, USAMRIID; positive control), or Synaptotagmin II_1–20_ (JPT, aa1–20, H-MRNIFKRNQEPIVAPATTTA-NH2; negative control). The competitor/inhibitor was added at concentrations of 2, 5, 20, 50, and 200 molar excess of BoNT/A. Cells were incubated with BoNT/A plus competitor/inhibitor for 2 hours. The toxin containing media was then removed and replaced with fresh media followed by overnight incubation. Cells lysates were analyzed by SDS-PAGE and Western blot analysis, using antibody against cleaved SNAP25, Anti-SNAP25_197_. Blots were quantified and the amount of SNAP25_197_ produced at each concentration, as a measure of BoNT/A uptake, was used to calculate the percent competition/inhibition. The amount of BoNT/A uptake for each competitor/inhibitor was compared to the amount of BoNT/A uptake after pre-treatment/pre-incubation with the negative control. Each experiment was conducted at least three independent times and each dose was tested in triplicates in each individual experiment, the percent average for each of the three or more independent experiments were used to generate inhibition curves. The curves were fitted to a non-linear exponential model; Y = 100×e −^b*log(concentration)^, where “b” was defined as either the competition constant (CC) or the inhibition constant (IC).

### Protein Complex Immunoprecipitation (Co-IP)

Differentiated Neuro-2a cells were washed with PBS and then lysed by incubation at 4°C for 30 minutes in lysis buffer containing 20 mM Tris, 150 mM NaCl, 1% Triton X-100, 1 mM EDTA, and 1 mM EGTA pH 7.2 plus complete protease inhibitor cocktail (11 873 580 001; Roche). The supernatant was collected by centrifugation and the total protein concentration was measured using Bradford Reagent (500-0205; Bio-Rad). The Co-IP reaction was performed by mixing 1 mg of cell lysate with 10 µg antibody in a total volume of 1 ml. The reaction was incubated at 4°C overnight. As negative controls, a sample without antibody (lysate only) and a sample without lysate (antibody only) were prepared in parallel. Then 100 µl Protein A/G Magnetic Beads (88802; Thermo Scientific) was added and the reaction was incubated at 4°C for 1 hour. Three times the beads were sedimented using a dynamag-2 magnet (Invitrogen; 12321D) and washed with PBS. Finally, the beads were re-suspended in 2× SDS-PAGE running buffer and Western Blot was performed. Cell lysate was run in parallel.

### Labeling of rH_C_/A and FGF2

Purified rH_C_/A (0.4 mg) was dialyzed against 4 L of 50 mM HEPES pH 7.0–7.2 150 mM NaCl in a 0.5 ml dialysis unit (Harvard Apparatus) for 16 hours at 4°C using a 25 kDa cut-off cellulose acetate membrane. FGF2 (0.2 mg) (233-FB/CF; R&D Systems) was re-suspended in 0.5 ml of 50 mM HEPES pH 7.2, 150 mM NaCl. rH_C_/A was labeled with either Alexa Fluor 488 C5-maleimide (A10254; Invitrogen) (cell imaging and photobleaching) or Alexa Fluor 633 C5-maleimide (A20342; Invitrogen) (rH_C_/A uptake) and FGF2 was labeled with Alexa Fluor 633 C5-maleimide (10∶1 molar ratio free label to protein) overnight at 4°C in the dark. To remove free label, the proteins were either dialyzed again, using the conditions listed above, or ran on a PD-10 desalting column (17-0851-01; GE Healthcare). The column was equilibrated with 50 mM HEPES pH 7.2 150 mM NaCl. The concentrations of rH_C_/A and FGF2 were determined by measuring the UV absorbance at 280 nm using a Beckman Coulter DU 800.

### Uptake of rH_C_/A and FGF2 in HEK 293 cells

Cells were plated at 20,000 cells per well in a Cell Carrier microplate (Perkin Elmer) in culture media and allowed to attach overnight. Cells were treated with 1 µg/ml Cell Tracker Green CMFDA (C2925; Invitrogen) and Hoechst 33342 (H10295; Invitrogen) for 30 minutes prior to adding fluorescently labeled rH_C_/A or FGF2. After removing the staining media, 0.1 ml of 0–25 nM of Alexa Fluor 633-rH_C_/A or -FGF2 was added. Fluorescence was measured using the Operetta High Content Imaging System (Perkin Elmer), set to the following parameters: 20× WD objective, 9 fields, non-confocal, 15% excitation, Blue (Ex 380–410/Em 430–460), Green (Ex 460–490 nm/Em 500–550 nm) and Far Red (Ex 630–645 nm/Em 660–900 nm). Uptake, measured as increasing amounts of Far Red signal in the cells, was monitored for 15 hours, with 30 minutes time points. The results were analyzed using Harmony 3.1 software.

### Cell imaging and photobleaching

PC-12 cells transfected with Halo tagged FGFR3 or SV2C were plated on Collagen IV coated glass bottom dishes (P35GCOL-0-10-C; MatTek) and differentiated for 3 days. The cells were incubated with 5 µM Halotag TMR ligand (G8251; Promega) for 15 minutes and washed 3 times for 10 minutes with fresh media. Cells were then treated with 1 µM Alexa Fluor 488 labeled rH_C_/A. After 2 hours incubation, the cells were fixed using 5% paraformaldehyde. Cells were imaged using a LSM710 confocal microscope and analyzed using ZEN 2009 software (Carl Zeiss INT, Germany). The Alexa Fluor 488 label and TMR star labels were imaged using the following respective settings: excitation 488 nm/emission 500–510 nm, excitation 561 nm/emission 595–620 nm. The TMR was photobleached by exciting the fluor with the 561 nm laser 100 times for 0.1 s with 5% laser power. The amount of fluorescence intensity of the donor fluor (AF-488) was measured before and after photobleaching of the acceptor (TMR).

### Immunostaining

Sprague-Dawley rats (200–250 g; Charles River) were injected with 10 units of BOTOX (Allergan) into the tibialis anterior (TA) muscle of the right hind limb. Animals receiving injections of 0.9% saline into their TA muscle served as controls. Rats were sacrificed 3 days following injections and their TA muscles were harvested. Muscles were embedded in OCT compound, frozen in liquid nitrogen and stored at −80°C. Prior to staining, muscles were cross-sectioned (10 µm) using a cryostat (Leica), mounted onto microscope slides and stored at −20°C until use. Frozen, slide-mounted muscle sections were thawed to room temperature and immediately fixed with 2% paraformaldehyde for 10 min. Sections were blocked with 5% normal serum in PBS, pH 7.4 for 60 minutes and then incubated with primary antibodies for 3 hours at room temperature: rabbit anti-SV2C (1∶400, sc-28957; Santa Cruz Biotechnology), rabbit anti-FGFR3 (1∶200, sc-123; Santa Cruz Biotechnology), mouse anti-SNAP25 (1∶200, SMI-81, Covance), and mouse anti-SNAP25_197_ (1∶200, Allergan). Muscle nicotinic acetylcholine receptors (nAChR) were labeled with α-bungarotoxin (α-Bgt) Alexa-Fluor 647 conjugate (1∶500, Invitrogen). Sections were then washed and incubated with secondary antibodies for 30 minutes at room temperature. Following a final wash, slides were coverslipped and analyzed. Images were acquired using a Zeiss LSM-710 confocal microscope (Carl Zeiss INT).

### Surface Plasmon Resonance (SPR) binding analysis

Experiments were performed on a BIAcore 3000 instrument (GE Healthcare). Ligands, rH_C_/A, anti-H_C_/A 6B1 (Provided by Dr. L. Smith, USAMRIID), FGF2, or FGF9 (233-FB; and 273-F9; R&D Systems), were immobilized on a CM5 chip (BR-1003-99, GE Healthcare) using an amine coupling kit (BR-1000-50, GE Healthcare). Analytes, either SV2C_529–579_ (JPT Peptide Technologies, dissolved in 100% DMSO), FGFR3b/c deletion mutant peptides (Figure 6A), rH_C_/A, or membrane extracts were injected over the ligand surfaces at concentrations ranging from 0–5000 nM, or for the membrane extractions at 5 µg/ml. The flow rate was set to 20 or 30 µl/min. Running buffer: HBS-EP buffer (BR-1006-91, GE Healthcare). The surfaces were re-generated by two 1-min injections at 30 µl/min of 10 mM Glycine, pH 1.5 (rH_C_/A) or 1-min injections at 30 µl/min of either; 10 mM Glycine, pH 1.5 and 0.125% SDS (FGFR3), 10 mM Glycine, pH 1.5 and 20 mM CHAPS (Membrane extracts), or 10 mM NaOH (SV2C). The sensorgram curves were evaluated using the BIAevaluation 3.0 software. The curves were fitted to a 1∶1 Langmuir binding model (A + B ↔ AB, where A is the analyte and B is the ligand immobilized on the sensor surface). Based on the fitted curves the association constant, k_a_, the dissociation constant, k_d_, and the equilibrium constant, K_D_ (K_D_ = k_d_/k_a_) were determined. The FGFR3b/c peptide curves were also visually compared using the “normalization” wizard in the BIAevaluation 4.1 software.

### T-test

To assess the significance of the differences from the BoNT/A cell-based competition/inhibition assays and the SPR Binding Analysis assays t-tests were performed using online Graphpad software; www.graphpad.com/quickcalcs/ttest1.cfm?Format=SEM (GraphPad Software Inc).

### ID numbers for genes and proteins mentioned in the text

FGFR3 (ENSG00000068078), SV2A (ENSG00000159164), SV2B (ENSG00000185518), SV2C (ENSG00000122012), FGF1 (ENSG00000113578), FGF2 (ENSG00000138685), FGF9 (ENSG00000102678), and FGF10 (ENSG00000070193).

## Supporting Information

Figure S1 **Isolation of a BoNT/A-FGFR3 protein complex and BoNT/A sensitivity and expression of SV2 and FGFR3 before and after differentiation.** (A) Undifferentiated and differentiated PC-12 cells were treated with BoNT/A for 6 hours followed by overnight incubation in toxin-free media. Western Blot of PC-12 cell lysates using antibodies specific to SNAP25_197_. Western blot band intensities were plotted against BoNT/A concentration in log scale using SigmaPlot v 10.0 and the EC_50_ values were calculated by fitting the curves to a 4-parameter logistics (4PL) function. PC-12 cells were more sensitive to BoNT/A after differentiation, observed as an increase in both potency and efficacy. The EC_50_ before differentiation was 800±20 pM, the EC_50_ after differentiation was 270±30 pM, and the efficacy of uptake was increased 2.5-fold (representative experiment of n = 3). (B) Three days differentiated Neuro-2a and PC-12 cells were treated with BoNT/A for 24 hours followed by two day incubation in toxin-free media. With longer treatment and incubation Neuro-2a and PC-12 cells were both very sensitive to BoNT/A. (Ba) Neuro-2a cells, EC_50_ = 60±5 pM and (Bb) PC-12 cells, EC_50_ = 47.1±13 pM (representative experiment of n≥4). Western Blot of cell lysates using antibodies specific to SNAP25_197_. Band intensities were plotted against BoNT/A concentration in log scale using SigmaPlot v 10.0 and fitted to a 4PL function. The SNAP25_197_ antibody is specific for the cleaved product and does not cross-react with SNAP25_206_ (No signal at the 0 pM concentration). (C–E) Isolation of a BoNT/A-FGFR3 protein complex in Neuro-2a cells. (C) Non-reducing SDS-PAGE, silver stained, showing the Avidin isolated ∼250 kDa protein complex from Neuro-2a cells treated with BoNT/A (150 kDa) carrying the cross-linking reagent Sulfo-SBED. As a negative control the pull down was performed with Sulfo-SBED BSA (Control). (D–E) Western blot under reducing conditions resulting in separation of BoNT/A and its receptor. The blot was probed with antibody against BoNT/A heavy chain (D) or FGFR3 (E) demonstrating that both BoNT/A and FGFR3 were present in the complex. The negative control with Sulfo-SBED BSA was run in parallel (Control). (F) Expression of FGFR3 and SV2 in Neuro-2a and PC-12 cells, before and after 3 days differentiation. Lysates of differentiated and non-differentiated cells were analyzed by Western blot using antibodies to FGFR3 and SV2A, B and C. The lysates, 6 µg per lane for FGFR3 and SV2A and B, and 3 µg per lane for SV2C, were run in parallel to allow direct comparison of intensities. According to the manufacturer the antibody to FGFR3 (Ab133644) recognizes a single band for FGFR3 just below 100 kDa. The antibodies to SV2 recognize multiple diffuse bands for SV2, partly because SV2 proteins are highly glycosylated in vivo [19]. The predicted size for each of them, based on their protein sequence, ∼80 kDa, is shown and an arrow is pointing to the band size expected to be SV2 for each lane. Interestingly, the band size for SV2 is higher in PC-12 than in Neuro-2a cells, suggesting different levels of glycosylation. As a loading control the blots we re-blotted using an antibody to Syntaxin. According to the manufacturer, the antibody for Syntaxin, sc-12736 recognizes a single band just above 34 kDa. Expression of FGFR3 was unchanged after differentiation, while the expression of SV2A, B and C was decreased in both cell lines. (G) Expression of FGFR3 and SV2 isoforms in HEK 293 cells. Lysates of HEK 293 cells (6 µg per lane) were analyzed by Western blot using antibodies to FGFR3 and SV2A, B and C. As a positive control, lysates from PC-12 cells were run in parallel. HEK 293 cells express FGFR3, but little or no SV2. (H) Uptake of rH_C_/A in HEK 293 cells. Uptake of 0–25 nM Alexa Fluor 633 labeled rH_C_/A (Hb) was measured, as an increase in intracellular fluorescence over time, using the Operetta High Content Imaging System. For comparison, internalization of Alexa Fluor 633 labeled FGF2 (Ha), a native ligand for FGFR3, was measured in parallel. The results show that rH_C_/A binds to and enters HEK 293 cells and that uptake of rH_C_/A is comparable to uptake of FGF2, even if slightly lower.
(TIF)

Figure S2 **Overexpression of FGFR3, but not SV2C, increases binding to rH_C_/A.** **Reduced expression of FGFR3 but not SV2C decreased sensitivity to BoNT/A.** (A–C) Affinity measurements with membrane extracts from PC-12 cells (A) or SH-SY5Y cells (B) transfected with FGFR3, SV2C, or empty vector (pcDNA3.1). Increased binding to rH_C_/A was observed using membrane extracts from cells transfected with FGFR3c compared to cells transfected with empty vector. No increase in response was observed using membrane extracts from cells transfected with SV2C compared to cells transfected with empty vector. (C) Table showing the average percentage increase in membrane extracts binding to rH_C_/A after transfection of PC-12, Neuro-2a, or SH-SY5Y cells with FGFR3c, compared to cells transfected with empty vector. (D–F) siRNA knockdown of FGFR3 or SV2C. Dose response curves of PC-12 cells transfected with Scrambled (negative control), FGFR3 siRNAs (D), or SV2C siRNA (E) and then treated with 0–30 nM BoNT/A for 6 hours. The curves were plotted as percent SNAP25 cleavage vs. BoNT/A concentration. (F) Table showing the calculated relative potency and 95% confidence interval, and fold-difference in mRNA and protein compared to the scrambled control for the FGFR3 and SV2C siRNAs used.
(TIF)

Figure S3 **FGFR3 Loop 2,3 is the minimal optimal binding site for rH_C_/A.** (A) Sequence alignment using Espript (from IBCP, Lyon, France) of the third extra-cellular loop of FGFR3 subtype b and c showing the amino acid sequence differences in loop 3 between the two subtypes. (B) Binding affinities of the eight FGFR3 deletion mutant peptides in a BIAcore SPR binding assay. Increasing concentrations, 0–4000 nM of each of the FGFR3 peptides were flowed across a CM5 sensor chip covered with rH_C_/A. The curves were fitted to a 1∶1 kinetic-binding model (A + B ↔ AB) with the BIAevaluation 3.0 software. The table shows the calculated k_a_ (1/Ms), k_d_ (1/s), and K_D_ (k_d_/k_a_ (M)) for all eight FGFR3 deletion mutant peptides upon binding to rH_C_/A. To assess the significance of the differences t-tests were performed, comparing each peptide to the longest peptide of the same subtype and comparing the subtype b peptides that were not significantly different from the longest FGFR3b peptide to the subtype c peptides that were not significantly different from the longest FGFR3c peptide. P-values are shown in the table. Unmarked p-values indicate that the difference is not significant. FGFR3b peptides bound slightly better than subtype c peptides. For both subtypes, the peptides that contained only Loop 3 bound with significantly lower affinity compared to the peptides that spanned Loop 2,3 or Loop 1,2,3. The average K_D_ for FGFR3c Loop 2,3 fell in the middle, being about 3 times higher than the average K_D_ for FGFR3c Loop 1,2,3. There was no significant difference in binding of FGFR3b Loop 2,3 and FGFR3b Loop 1,2,3, showing that Loop 2,3 of FGFR3 is the minimal optimal binding region for rH_C_/A. (C) Visual comparison of FGFR3c Loop 1,2,3 , FGFR3c Loop 2,3, and FGFR3c Loop 3. The 1000 nM curves of one or two runs for each peptide were normalized using BIAcore evaluation software version 4.1. The on-rate of FGFR3c Loop 1,2,3 and FGFR3c Loop 2,3 are faster than the on-rate for FGFR3c Loop 3.
(TIF)

## References

[1] Blasi J, Chapman ER, Link E, Binz T, Yamasaki S, et al (1993) Botulinum neurotoxin A selectively cleaves the synaptic protein SNAP-25. Nature 365: 160–163 10.1038/365160a0 [doi]. 8103915 10.1038/365160a0

[2] Montecucco C, Papini E, Schiavo G (1994) Bacterial protein toxins penetrate cells via a four-step mechanism. FEBS Lett 346: 92–98. 8206166 10.1016/0014-5793(94)00449-8

[3] Keller JE, Cai F, Neale EA (2004) Uptake of botulinum neurotoxin into cultured neurons. Biochemistry 43: 526–532. 14717608 10.1021/bi0356698

[4] Verderio C, Grumelli C, Raiteri L, Coco S, Paluzzi S, et al (2007) Traffic of botulinum toxins A and E in excitatory and inhibitory neurons. Traffic 8: 142–153. 17241445 10.1111/j.1600-0854.2006.00520.x

[5] Popoff MR, Poulain B (2010) Bacterial Toxins and the Nervous System: Neurotoxins and Multipontetial Toxins Interacting with Neuronal Cells. Toxins 2: 683–737. 22069606 10.3390/toxins2040683 PMC3153206

[6] Schulte-Mattler WJ (2008) Use of botulinum toxin A in adult neurological disorders: efficacy, tolerability and safety. CNS Drugs 22: 725–738. 18698873 10.2165/00023210-200822090-00002

[7] Apostolidis A, Fowler CJ (2008) The use of botulinum neurotoxin type A (BoNTA) in urology. J Neural Transm 115: 593–605. 18322639 10.1007/s00702-007-0862-x

[8] Montal M (2010) Botulinum Neurotoxin: A Marvel of Protein Design. Annual Review of Biochemistry 79: 591–617. 10.1146/annurev.biochem.051908.125345 20233039

[9] Burstein R, Dodick D, Silberstein S (2009) Migraine prophylaxis with botulinum toxin A is associated with perception of headache. Toxicon 54: 624–627 S0041-0101(09)00042-7 [pii];10.1016/j.toxicon.2009.01.009 [doi]. 19344670 10.1016/j.toxicon.2009.01.009 PMC2731008

[10] Schieman C, Gelfand GJ, Grondin SC (2010) Hyperhidrosis: clinical presentation, evaluation and management. Expert Rev Dermatol 5: 31–44.

[11] Dolly JO, Black J, Williams RS, Melling J (1984) Acceptors for botulinum neurotoxin reside on motor nerve terminals and mediate its internalization. Nature 307: 457–460. 6694738 10.1038/307457a0

[12] Montecucco C (1986) How do Tetanus and Botulinum toxins to neuronal membranes? Trends Biochem Sci 11: 315–317.

[13] Montecucco C, Rossetto O, Schiavo G (2004) Presynaptic receptor arrays for clostridial neurotoxins. Trends Microbiol 12: 442–446. 15381192 10.1016/j.tim.2004.08.002

[14] Rummel A, Mahrhold S, Bigalke H, Binz T (2004) The HCC-domain of botulinum neurotoxins A and B exhibits a singular ganglioside binding site displaying serotype specific carbohydrate interaction. Mol Microbiol 51: 631–643. 14731268 10.1046/j.1365-2958.2003.03872.x

[15] Stenmark P, Dupuy J, Imamura A, Kiso M, Stevens RC (2008) Crystal structure of botulinum neurotoxin type A in complex with the cell surface co-receptor GT1b-insight into the toxin-neuron interaction. PLoS Pathog 4: e1000129 10.1371/journal.ppat.1000129 [doi]. 18704164 10.1371/journal.ppat.1000129 PMC2493045

[16] Yowler BC, Schengrund CL (2004) Botulinum neurotoxin A changes conformation upon binding to ganglioside GT1b. Biochemistry 43: 9725–9731. 15274627 10.1021/bi0494673

[17] Mahrhold S, Rummel A, Bigalke H, Davletov B, Binz T (2006) The synaptic vesicle protein 2C mediates the uptake of botulinum neurotoxin A into phrenic nerves. FEBS Lett 580: 2011–2014. 16545378 10.1016/j.febslet.2006.02.074

[18] Dong M, Yeh F, Tepp WH, Dean C, Johnson EA, et al (2006) SV2 is the protein receptor for botulinum neurotoxin A. Science 312: 592–596. 16543415 10.1126/science.1123654

[19] Verderio C, Rossetto O, Grumelli C, Frassoni C, Montecucco C, et al (2006) Entering neurons: botulinum toxins and synaptic vesicle recycling. EMBO Rep 7: 995–999. 17016457 10.1038/sj.embor.7400796 PMC1618376

[20] Dong M, Liu H, Tepp WH, Johnson EA, Janz R, et al (2008) Glycosylated SV2A and SV2B mediate the entry of botulinum neurotoxin E into neurons. Mol Biol Cell 19: 5226–5237. 18815274 10.1091/mbc.E08-07-0765 PMC2592654

[21] Baldwin MR, Barbieri JT (2009) Association of botulinum neurotoxins with synaptic vesicle protein complexes. Toxicon 54: 570–574. 19362106 10.1016/j.toxicon.2009.01.040 PMC2730980

[22] Rummel A, Hafner K, Mahrhold S, Darashchonak N, Holt M, et al (2009) Botulinum neurotoxins C, E and F bind gangliosides via a conserved binding site prior to stimulation-dependent uptake with botulinum neurotoxin F utilising the three isoforms of SV2 as second receptor. J Neurochem 110: 1942–1954. 19650874 10.1111/j.1471-4159.2009.06298.x

[23] Rummel A (2013) Double Receptor Anchorage of Botulinum Neurotoxins Accounts for their Exquisite Neurospecificity. Curr Top Microbiol Immunol 364: 61–90 10.1007/978-3-642-33570-9_4 [doi]. 23239349 10.1007/978-3-642-33570-9_4

[24] Fu Z, Chen C, Barbieri JT, Kim JJ, Baldwin MR (2009) Glycosylated SV2 and gangliosides as dual receptors for botulinum neurotoxin serotype F. Biochemistry 48: 5631–5641. 19476346 10.1021/bi9002138 PMC2709598

[25] Strotmeier J, Lee K, Volker AK, Mahrhold S, Zong Y, et al (2010) Botulinum neurotoxin serotype D attacks neurons via two carbohydrate-binding sites in a ganglioside-dependent manner. Biochem J 431: 207–216 BJ20101042 [pii];10.1042/BJ20101042 [doi]. 20704566 10.1042/BJ20101042

[26] Peng L, Tepp WH, Johnson EA, Dong M (2011) Botulinum Neurotoxin D Uses Synaptic Vesicle Protein SV2 and Gangliosides as Receptors. PLoS Pathog 7: e1002008 10.1371/journal.ppat.1002008 [doi]. 21483489 10.1371/journal.ppat.1002008 PMC3068998

[27] Yeh FL, Dong M, Yao J, Tepp WH, Lin G, et al (2010) SV2 mediates entry of tetanus neurotoxin into central neurons. PLoS Pathog 6: e1001207 10.1371/journal.ppat.1001207 [doi]. 21124874 10.1371/journal.ppat.1001207 PMC2991259

[28] Lacy DB, Tepp W, Cohen AC, DasGupta BR, Stevens RC (1998) Crystal structure of botulinum neurotoxin type A and implications for toxicity. Nat Struct Biol 5: 898–902. 9783750 10.1038/2338

[29] Lacy DB, Stevens RC (1999) Sequence homology and structural analysis of the clostridial neurotoxins. J Mol Biol 291: 1091–1104 S0022-2836(99)92945-5 [pii];10.1006/jmbi.1999.2945 [doi]. 10518945 10.1006/jmbi.1999.2945

[30] Keegan K, Johnson DE, Williams LT, Hayman MJ (1991) Isolation of an additional member of the fibroblast growth factor receptor family, FGFR-3. Proc Natl Acad Sci U S A 88: 1095–1099. 1847508 10.1073/pnas.88.4.1095 PMC50963

[31] Keegan K, Meyer S, Hayman MJ (1991) Structural and biosynthetic characterization of the fibroblast growth factor receptor 3 (FGFR-3) protein. Oncogene 6: 2229–2236. 1662791

[32] Webster MK, Donoghue DJ (1996) Constitutive activation of fibroblast growth factor receptor 3 by the transmembrane domain point mutation found in achondroplasia. EMBO J 15: 520–527. 8599935 PMC449970

[33] Webster MK, D'Avis PY, Robertson SC, Donoghue DJ (1996) Profound ligand-independent kinase activation of fibroblast growth factor receptor 3 by the activation loop mutation responsible for a lethal skeletal dysplasia, thanatophoric dysplasia type II. Mol Cell Biol 16: 4081–4087. 8754806 10.1128/mcb.16.8.4081 PMC231404

[34] Hart KC, Robertson SC, Kanemitsu MY, Meyer AN, Tynan JA, et al (2000) Transformation and Stat activation by derivatives of FGFR1, FGFR3, and FGFR4. Oncogene 19: 3309–3320 10.1038/sj.onc.1203650 [doi]. 10918587 10.1038/sj.onc.1203650

[35] Beenken A, Mohammadi M (2009) The FGF family: biology, pathophysiology and therapy. Nat Rev Drug Discov 8: 235–253 nrd2792 [pii];10.1038/nrd2792 [doi]. 19247306 10.1038/nrd2792 PMC3684054

[36] Johnson DE, Lu J, Chen H, Werner S, Williams LT (1991) The human fibroblast growth factor receptor genes: a common structural arrangement underlies the mechanisms for generating receptor forms that differ in their third immunoglobulin domain. Mol Cell Biol 11: 4627–4634. 1652059 10.1128/mcb.11.9.4627-4634.1991 PMC361347

[37] Werner S, Duan DS, de VC, Peters KG, Johnson DE, Williams LT (1992) Differential splicing in the extracellular region of fibroblast growth factor receptor 1 generates receptor variants with different ligand-binding specificities. Mol Cell Biol 12: 82–88. 1309595 10.1128/mcb.12.1.82-88.1992 PMC364071

[38] Chellaiah AT, McEwen DG, Werner S, Xu J, Ornitz DM (1994) Fibroblast growth factor receptor (FGFR) 3. Alternative splicing in immunoglobulin-like domain III creates a receptor highly specific for acidic FGF/FGF-1. J Biol Chem 269: 11620–11627. 7512569

[39] Zhang X, Ibrahimi OA, Olsen SK, Umemori H, Mohammadi M, et al (2006) Receptor specificity of the fibroblast growth factor family. The complete mammalian FGF family. J Biol Chem 281: 15694–15700. 16597617 10.1074/jbc.M601252200 PMC2080618

[40] Sturla LM, Merrick AE, Burchill SA (2003) FGFR3IIIS: a novel soluble FGFR3 spliced variant that modulates growth is frequently expressed in tumour cells. Br J Cancer 89: 1276–1284 10.1038/sj.bjc.6601249 [doi];6601249 [pii]. 14520460 10.1038/sj.bjc.6601249 PMC2394287

[41] Champion-Arnaud P, Ronsin C, Gilbert E, Gesnel MC, Houssaint E, et al (1991) Multiple mRNAs code for proteins related to the BEK fibroblast growth factor receptor. Oncogene 6: 979–987. 1648704

[42] Ornitz DM, Xu J, Colvin JS, McEwen DG, MacArthur CA, et al (1996) Receptor specificity of the fibroblast growth factor family. J Biol Chem 271: 15292–15297. 8663044 10.1074/jbc.271.25.15292

[43] Rusnati M, Urbinati C, Tanghetti E, Dell'Era P, Lortat-Jacob H, et al (2002) Cell membrane GM1 ganglioside is a functional coreceptor for fibroblast growth factor 2. Proc Natl Acad Sci U S A 99: 4367–4372. 11917140 10.1073/pnas.072651899 PMC123654

[44] Toledo MS, Suzuki E, Handa K, Hakomori S (2005) Effect of ganglioside and tetraspanins in microdomains on interaction of integrins with fibroblast growth factor receptor. J Biol Chem 280: 16227–16234. 15710618 10.1074/jbc.M413713200

[45] Eswarakumar VP, Lax I, Schlessinger J (2005) Cellular signaling by fibroblast growth factor receptors. Cytokine Growth Factor Rev 16: 139–149 S1359-6101(05)00002-X [pii];10.1016/j.cytogfr.2005.01.001 [doi]. 15863030 10.1016/j.cytogfr.2005.01.001

[46] West DC, Rees CG, Duchesne L, Patey SJ, Terry CJ, et al (2005) Interactions of multiple heparin binding growth factors with neuropilin-1 and potentiation of the activity of fibroblast growth factor-2. J Biol Chem 280: 13457–13464. 15695515 10.1074/jbc.M410924200

[47] Kurosu H, Ogawa Y, Miyoshi M, Yamamoto M, Nandi A, et al (2006) Regulation of fibroblast growth factor-23 signaling by klotho. J Biol Chem 281: 6120–6123. 16436388 10.1074/jbc.C500457200 PMC2637204

[48] Hu Y, Guimond SE, Travers P, Cadman S, Hohenester E, et al (2009) Novel mechanisms of fibroblast growth factor receptor 1 regulation by extracellular matrix protein anosmin-1. J Biol Chem 284: 29905–29920. 19696444 10.1074/jbc.M109.049155 PMC2785620

[49] Greene LA, Tischler AS (1976) Establishment of a noradrenergic clonal line of rat adrenal pheochromocytoma cells which respond to nerve growth factor. Proc Natl Acad Sci U S A 73: 2424–2428. 1065897 10.1073/pnas.73.7.2424 PMC430592

[50] Puffer EB, Lomneth RB, Sarkar HK, Singh BR (2001) Differential roles of developmentally distinct SNAP-25 isoforms in the neurotransmitter release process. Biochemistry 40: 9374–9378. 11478906 10.1021/bi010362z

[51] Olsen SK, Garbi M, Zampieri N, Eliseenkova AV, Ornitz DM, et al (2003) Fibroblast growth factor (FGF) homologous factors share structural but not functional homology with FGFs. J Biol Chem 278: 34226–34236 10.1074/jbc.M303183200 [doi];M303183200 [pii]. 12815063 10.1074/jbc.M303183200

[52] Olsen SK, Ibrahimi OA, Raucci A, Zhang F, Eliseenkova AV, et al (2004) Insights into the molecular basis for fibroblast growth factor receptor autoinhibition and ligand-binding promiscuity. Proc Natl Acad Sci U S A 101: 935–940. 14732692 10.1073/pnas.0307287101 PMC327120

[53] Nishiki T, Kamata Y, Nemoto Y, Omori A, Ito T, et al (1994) Identification of protein receptor for Clostridium botulinum type B neurotoxin in rat brain synaptosomes. J Biol Chem 269: 10498–10503. 8144634

[54] Dong M, Richards DA, Goodnough MC, Tepp WH, Johnson EA, et al (2003) Synaptotagmins I and II mediate entry of botulinum neurotoxin B into cells. J Cell Biol 162: 1293–1303 10.1083/jcb.200305098 [doi];jcb.200305098 [pii]. 14504267 10.1083/jcb.200305098 PMC2173968

[55] Chai Q, Arndt JW, Dong M, Tepp WH, Johnson EA, et al (2006) Structural basis of cell surface receptor recognition by botulinum neurotoxin B. Nature 444: 1096–1100 nature05411 [pii];10.1038/nature05411 [doi]. 17167418 10.1038/nature05411

[56] Jin R, Rummel A, Binz T, Brunger AT (2006) Botulinum neurotoxin B recognizes its protein receptor with high affinity and specificity. Nature 444: 1092–1095. 17167421 10.1038/nature05387

[57] Li S, Christensen C, Kohler LB, Kiselyov VV, Berezin V, et al (2009) Agonists of fibroblast growth factor receptor induce neurite outgrowth and survival of cerebellar granule neurons. Dev Neurobiol 69: 837–854 10.1002/dneu.20740 [doi]. 19634127 10.1002/dneu.20740

[58] Purkiss JR, Friis LM, Doward S, Quinn CP (2001) Clostridium botulinum neurotoxins act with a wide range of potencies on SH-SY5Y human neuroblastoma cells. Neurotoxicology 22: 447–453. 11577803 10.1016/s0161-813x(01)00042-0

[59] Wiedlocha A, Sorensen V (2004) Signaling, internalization, and intracellular activity of fibroblast growth factor. Curr Top Microbiol Immunol 286: 45–79. 15645710 10.1007/978-3-540-69494-6_3

[60] Hughes R, Whaler BC (1962) Influence of nerve-ending activity and of drugs on the rate of paralysis of rat diaphragm preparations by Cl. botulinum type A toxin. J Physiol 160: 221–233. 14449824 10.1113/jphysiol.1962.sp006843 PMC1359529

[61] Habermann E, Dreyer F, Bigalke H (1980) Tetanus toxin blocks the neuromuscular transmission in vitro like botulinum A toxin. Naunyn Schmiedebergs Arch Pharmacol 311: 33–40. 6245375 10.1007/BF00500299

[62] Black JD, Dolly JO (1986) Interaction of 125I-labeled botulinum neurotoxins with nerve terminals. II. Autoradiographic evidence for its uptake into motor nerves by acceptor-mediated endocytosis. J Cell Biol 103: 535–544. 3015983 10.1083/jcb.103.2.535 PMC2113823

[63] Restani L, Giribaldi F, Manich M, Bercsenyi K, Menendez G, et al (2012) Botulinum neurotoxins a and e undergo retrograde axonal transport in primary motor neurons. PLoS Pathog 8: e1003087 10.1371/journal.ppat.1003087 [doi];PPATHOGENS-D-12-00849 [pii]. 23300443 10.1371/journal.ppat.1003087 PMC3531519

[64] Simpson LL (1980) Kinetic studies on the interaction between botulinum toxin type A and the cholinergic neuromuscular junction. J Pharmacol Exp Ther 212: 16–21. 6243359

[65] Duchesne L, Octeau V, Bearon RN, Beckett A, Prior IA, et al (2012) Transport of fibroblast growth factor 2 in the pericellular matrix is controlled by the spatial distribution of its binding sites in heparan sulfate. PLoS Biol 10: e1001361 10.1371/journal.pbio.1001361 [doi];PBIOLOGY-D-11-03009 [pii]. 22815649 10.1371/journal.pbio.1001361 PMC3398970

[66] Yowler BC, Kensinger RD, Schengrund CL (2002) Botulinum neurotoxin A activity is dependent upon the presence of specific gangliosides in neuroblastoma cells expressing synaptotagmin I. J Biol Chem 277: 32815–32819. 12089155 10.1074/jbc.M205258200

[67] Kitamura M, Takamiya K, Aizawa S, Furukawa K, Furukawa K (1999) Gangliosides are the binding substances in neural cells for tetanus and botulinum toxins in mice. Biochim Biophys Acta 1441: 1–3 S1388-1981(99)00140-7 [pii]. 10526222 10.1016/s1388-1981(99)00140-7

[68] Elias M, Al-Saleem F, Ancharski DM, Singh A, Nasser Z, et al (2011) Evidence that botulinum toxin receptors on epithelial cells and neuronal cells are not identical: implications for development of a non-neurotropic vaccine. J Pharmacol Exp Ther 336: 605–612 jpet.110.175018 [pii];10.1124/jpet.110.175018 [doi]. 21106906 10.1124/jpet.110.175018 PMC3061530

[69] Wuechner C, Nordqvist AC, Winterpacht A, Zabel B, Schalling M (1996) Developmental expression of splicing variants of fibroblast growth factor receptor 3 (FGFR3) in mouse. Int J Dev Biol 40: 1185–1188. 9032024

[70] Fon TK, Bookout AL, Ding X, Kurosu H, John GB, et al (2010) Research resource: Comprehensive expression atlas of the fibroblast growth factor system in adult mouse. Mol Endocrinol 24: 2050–2064 me.2010-0142 [pii];10.1210/me.2010-0142 [doi]. 20667984 10.1210/me.2010-0142 PMC2954642

[71] Bajjalieh SM, Peterson K, Linial M, Scheller RH (1993) Brain contains two forms of synaptic vesicle protein 2. Proc Natl Acad Sci U S A 90: 2150–2154. 7681585 10.1073/pnas.90.6.2150 PMC46043

[72] Janz R, Sudhof TC (1999) SV2C is a synaptic vesicle protein with an unusually restricted localization: anatomy of a synaptic vesicle protein family. Neuroscience 94: 1279–1290. 10625067 10.1016/s0306-4522(99)00370-x

[73] Mohammadi M, Olsen SK, Ibrahimi OA (2005) Structural basis for fibroblast growth factor receptor activation. Cytokine Growth Factor Rev 16: 107–137. 15863029 10.1016/j.cytogfr.2005.01.008

