# Supplementary figures and images for: Identification of Fibroblast Growth Factor Receptor 3 (FGFR3) as a Protein Receptor for Botulinum Neurotoxin Serotype A (BoNT/A)
Source: PLoS Pathog. 2013 May 16;9(5):e1003369. doi: 10.1371/journal.ppat.1003369 (PMC3656097; doi:10.1371/journal.ppat.1003369)

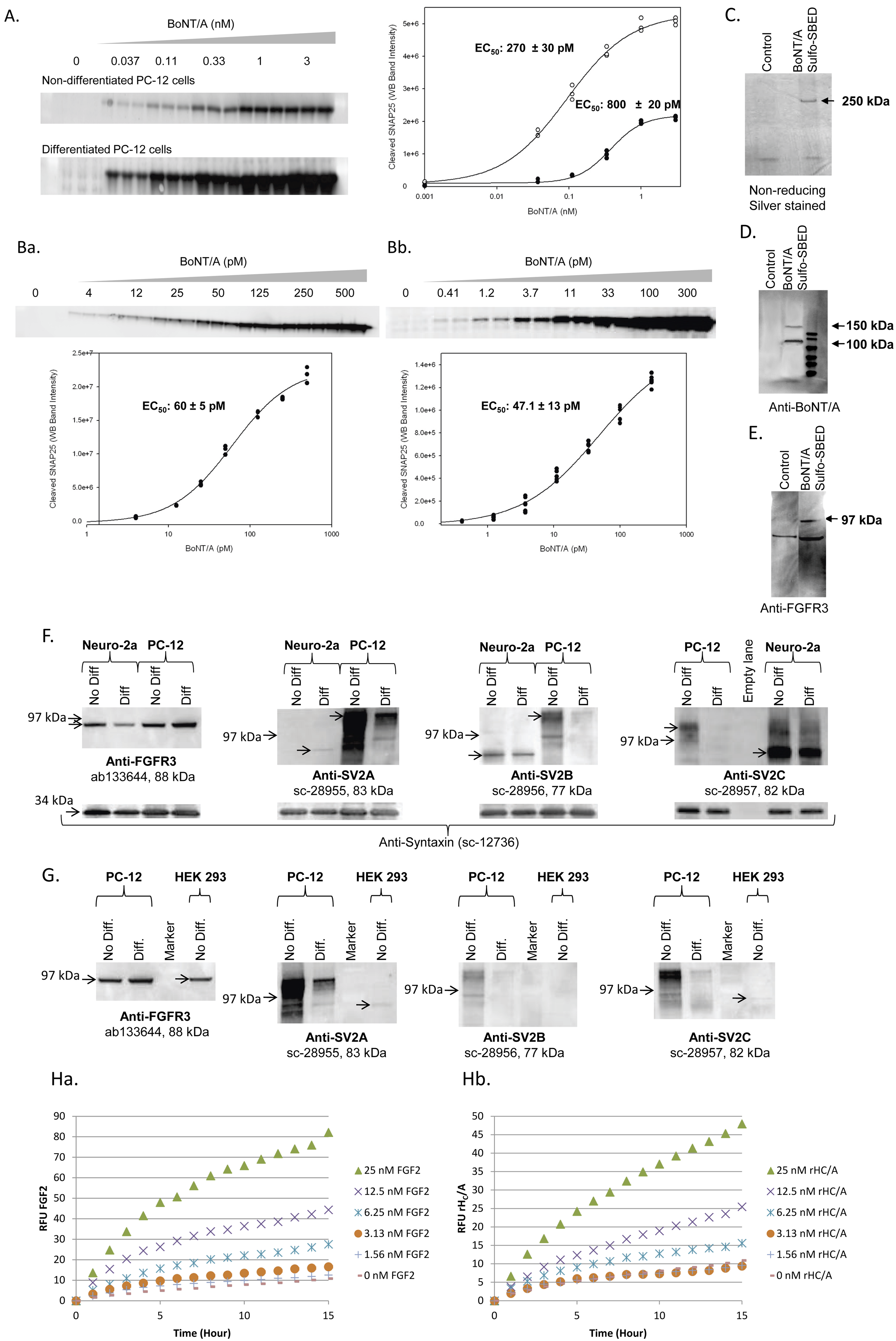

Supplement: Figure S1 — Isolation of a BoNT/A-FGFR3 protein complex and BoNT/A sensitivity and expression of SV2 and FGFR3 before and after differentiation. (A) Undifferentiated and differentiated PC-12 cells were treated with BoNT/A for 6 hours followed by overnight incubation in toxin-free media. Western Blot of PC-12 cell lysates using antibodies specific to SNAP25197. Western blot band intensities were plotted against BoNT/A concentration in log scale using SigmaPlot v 10.0 and the EC50 values were calculated by fitting the curves to a 4-parameter logistics (4PL) function. PC-12 cells were more sensitive to BoNT/A after differentiation, observed as an increase in both potency and efficacy. The EC50 before differentiation was 800±20 pM, the EC50 after differentiation was 270±30 pM, and the efficacy of uptake was increased 2.5-fold (representative experiment of n = 3). (B) Three days differentiated Neuro-2a and PC-12 cells were treated with BoNT/A for 24 hours followed by two day incubation in toxin-free media. With longer treatment and incubation Neuro-2a and PC-12 cells were both very sensitive to BoNT/A. (Ba) Neuro-2a cells, EC50 = 60±5 pM and (Bb) PC-12 cells, EC50 = 47.1±13 pM (representative experiment of n≥4). Western Blot of cell lysates using antibodies specific to SNAP25197. Band intensities were plotted against BoNT/A concentration in log scale using SigmaPlot v 10.0 and fitted to a 4PL function. The SNAP25197 antibody is specific for the cleaved product and does not cross-react with SNAP25206 (No signal at the 0 pM concentration). (C–E) Isolation of a BoNT/A-FGFR3 protein complex in Neuro-2a cells. (C) Non-reducing SDS-PAGE, silver stained, showing the Avidin isolated ∼250 kDa protein complex from Neuro-2a cells treated with BoNT/A (150 kDa) carrying the cross-linking reagent Sulfo-SBED. As a negative control the pull down was performed with Sulfo-SBED BSA (Control). (D–E) Western blot under reducing conditions resulting in separation of BoNT/A and its receptor. The blot was [file ppat.1003369.s001.tif]

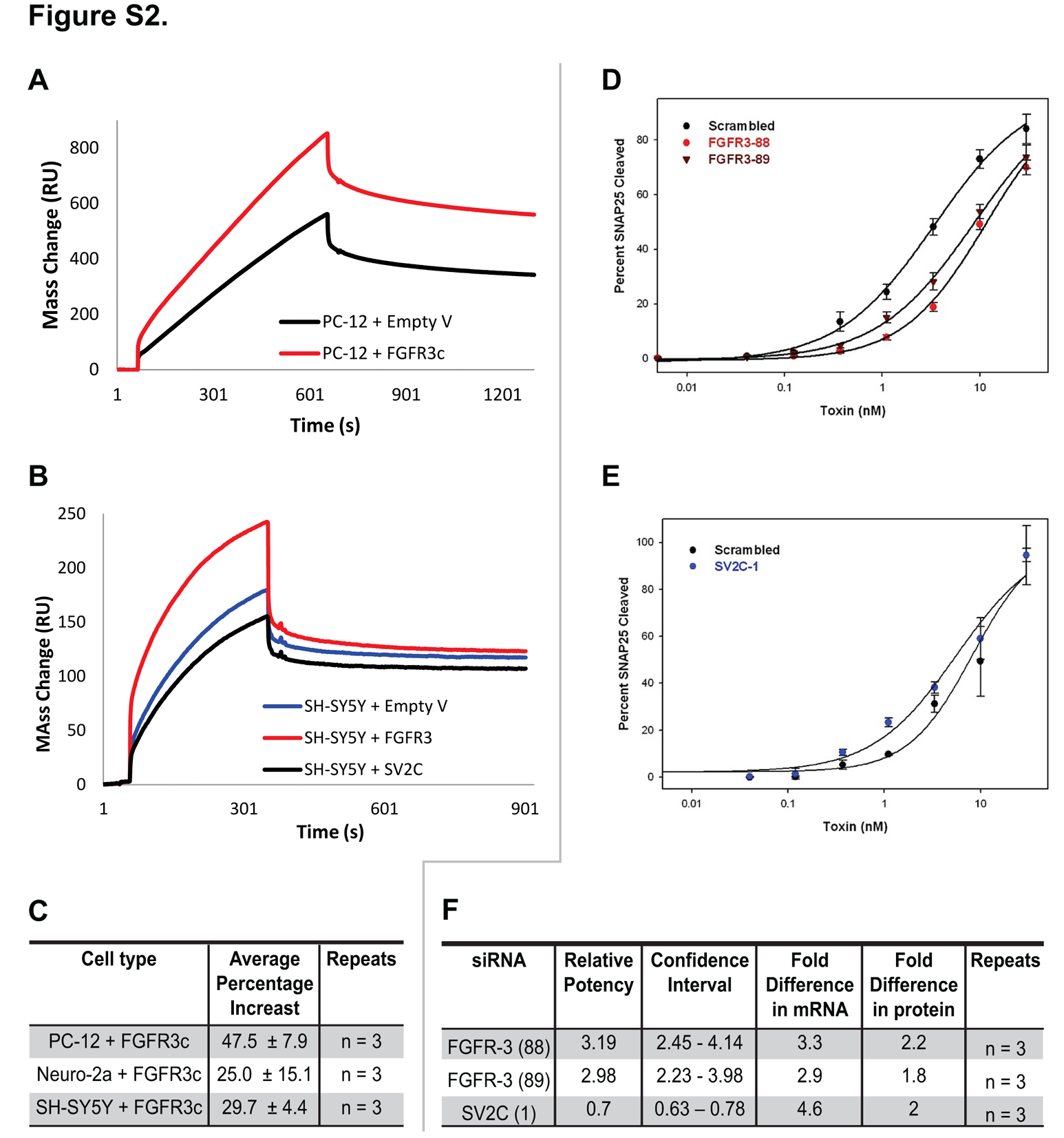

Supplement: Figure S2 — Overexpression of FGFR3, but not SV2C, increases binding to rHC/A. Reduced expression of FGFR3 but not SV2C decreased sensitivity to BoNT/A. (A–C) Affinity measurements with membrane extracts from PC-12 cells (A) or SH-SY5Y cells (B) transfected with FGFR3, SV2C, or empty vector (pcDNA3.1). Increased binding to rHC/A was observed using membrane extracts from cells transfected with FGFR3c compared to cells transfected with empty vector. No increase in response was observed using membrane extracts from cells transfected with SV2C compared to cells transfected with empty vector. (C) Table showing the average percentage increase in membrane extracts binding to rHC/A after transfection of PC-12, Neuro-2a, or SH-SY5Y cells with FGFR3c, compared to cells transfected with empty vector. (D–F) siRNA knockdown of FGFR3 or SV2C. Dose response curves of PC-12 cells transfected with Scrambled (negative control), FGFR3 siRNAs (D), or SV2C siRNA (E) and then treated with 0–30 nM BoNT/A for 6 hours. The curves were plotted as percent SNAP25 cleavage vs. BoNT/A concentration. (F) Table showing the calculated relative potency and 95% confidence interval, and fold-difference in mRNA and protein compared to the scrambled control for the FGFR3 and SV2C siRNAs used. (TIF) [file ppat.1003369.s002.tif]

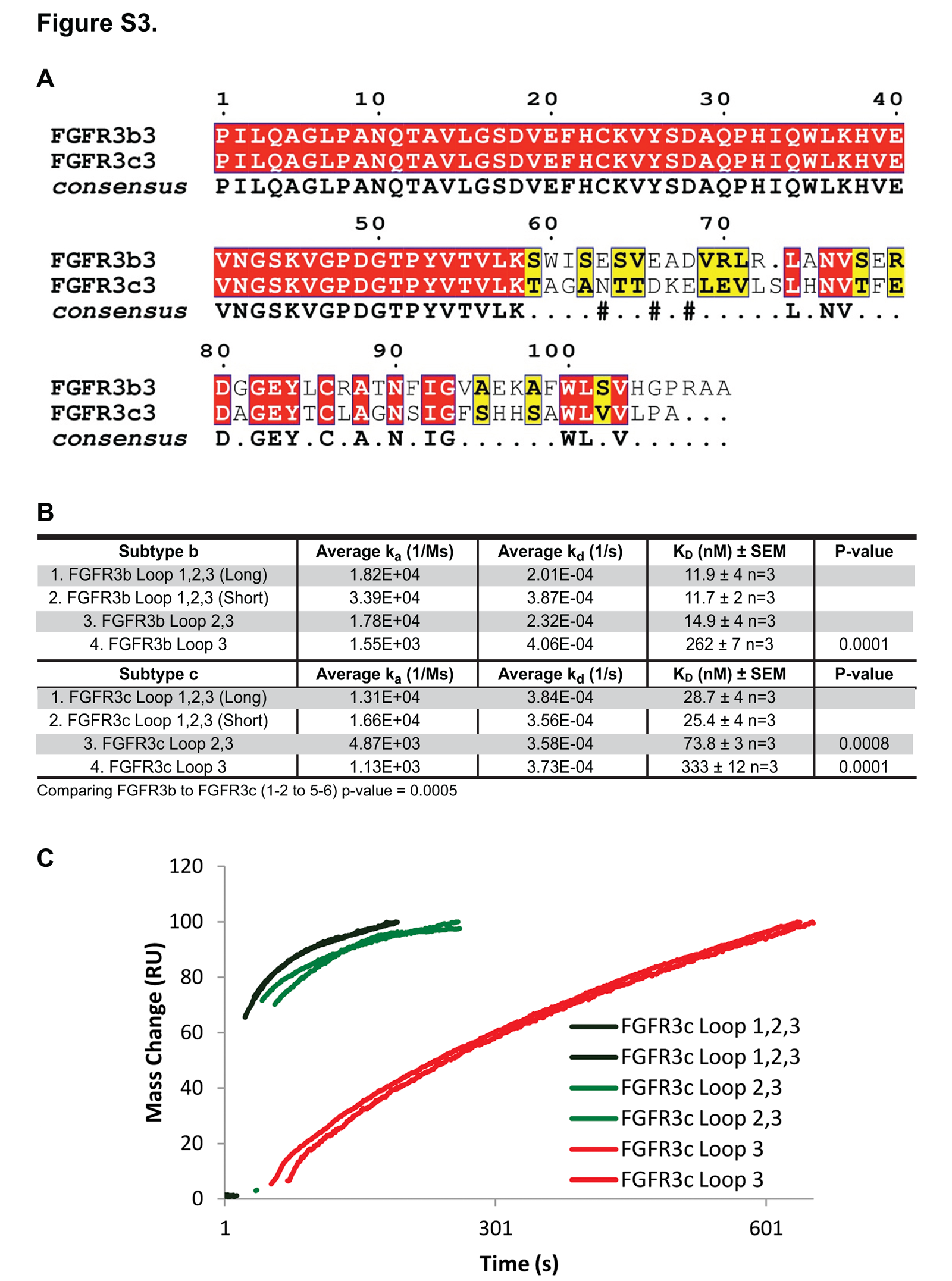

Supplement: Figure S3 — FGFR3 Loop 2,3 is the minimal optimal binding site for rHC/A. (A) Sequence alignment using Espript (from IBCP, Lyon, France) of the third extra-cellular loop of FGFR3 subtype b and c showing the amino acid sequence differences in loop 3 between the two subtypes. (B) Binding affinities of the eight FGFR3 deletion mutant peptides in a BIAcore SPR binding assay. Increasing concentrations, 0–4000 nM of each of the FGFR3 peptides were flowed across a CM5 sensor chip covered with rHC/A. The curves were fitted to a 1∶1 kinetic-binding model (A + B ↔ AB) with the BIAevaluation 3.0 software. The table shows the calculated ka (1/Ms), kd (1/s), and KD (kd/ka (M)) for all eight FGFR3 deletion mutant peptides upon binding to rHC/A. To assess the significance of the differences t-tests were performed, comparing each peptide to the longest peptide of the same subtype and comparing the subtype b peptides that were not significantly different from the longest FGFR3b peptide to the subtype c peptides that were not significantly different from the longest FGFR3c peptide. P-values are shown in the table. Unmarked p-values indicate that the difference is not significant. FGFR3b peptides bound slightly better than subtype c peptides. For both subtypes, the peptides that contained only Loop 3 bound with significantly lower affinity compared to the peptides that spanned Loop 2,3 or Loop 1,2,3. The average KD for FGFR3c Loop 2,3 fell in the middle, being about 3 times higher than the average KD for FGFR3c Loop 1,2,3. There was no significant difference in binding of FGFR3b Loop 2,3 and FGFR3b Loop 1,2,3, showing that Loop 2,3 of FGFR3 is the minimal optimal binding region for rHC/A. (C) Visual comparison of FGFR3c Loop 1,2,3 , FGFR3c Loop 2,3, and FGFR3c Loop 3. The 1000 nM curves of one or two runs for each peptide were normalized using BIAcore evaluation software version 4.1. The on-rate of FGFR3c Loop 1,2,3 and FGFR3c Loop 2,3 are faster than the on-rate for FGFR3c Loop 3. (TIF) [file ppat.1003369.s003.tif]
